# Tumor size limits macrophage activation and imaging response to CD47 blockade in osteosarcoma

**DOI:** 10.1016/j.biopha.2026.119846

**Published:** 2026-08-17

**Authors:** Raheleh Roudi, Giovanni Marco Saladino, Iryna Vasyliv, Laura Pisani, Jason Wong, Wencke Reineking, Ramesh Duwa, Allison Pribnow, Heike E. Daldrup-Link

**Affiliations:** a Department of Radiology, Molecular Imaging Program at Stanford, Stanford University School of Medicine, Stanford, CA, USA; b Department of Comparative Medicine, Stanford University School of Medicine, Stanford, CA, USA; c Department of Pediatrics, Hematology/Oncology, Stanford University School of Medicine, Stanford, CA, USA

**Keywords:** CD47, Immunotherapy, MRI, Osteosarcoma, Ferumoxytol, Iron oxide nanoparticle

## Abstract

Despite robust preclinical activity, CD47-targeting agents demonstrate limited single-agent efficacy in osteosarcomas. The purpose of our study was to investigate whether tumor size at baseline affects tumor-associated macrophage (TAM) response to CD47 mAb, as measured by ferumoxytol-MRI. Thirty female NOD SCID gamma mice and thirty BALB/c mice with small, medium-sized, and large 143B osteosarcoma xenografts or murine K7M2 tumors were treated with either CD47 mAb or PBS. All mice underwent MRI scans at baseline, one week after treatment, and after intravenous infusion of ferumoxytol nanoparticles. We measured tumor T2* relaxation times as a quantitative measure of nanoparticle retention in activated TAMs. Tumor ΔT2* was calculated as the difference between T2* post-treatment pre-contrast and T2* post-treatment post-contrast to quantify ferumoxytol enhancement. T2* and ΔT2* values were compared between treatment groups using regression models including treatment, tumor size, and their interaction. Histology served as a standard of reference. At baseline, tumors in CD47 mAb- and PBS-treated mice demonstrated no significant differences in T2* relaxation time, regardless of tumor size (all p > 0.05). After CD47 mAb therapy and ferumoxytol infusion, tumors treated with CD47 mAb demonstrated significantly shorter T2* relaxation times compared with PBS-treated tumors (all p < 0.001). The tumor ΔT2* enhancement was significantly higher in small tumors compared with large tumors (all p < 0.001), indicating higher nanoparticle accumulation in small tumors after CD47 blockade. Histopathology showed a significantly higher F4/80^+^CD80^+^ TAM staining in CD47 mAb-treated small tumors compared with large tumors (all p < 0.05). In conclusion, tumor size at baseline affects TAM response to CD47 mAb. Smaller osteosarcomas exhibit a more pronounced TAM response to CD47 mAb therapy compared with larger tumors.

## 1. Introduction

Osteosarcoma is the most common and most aggressive bone tumor in children and young adults, with five-year survival rates below 20% in patients with metastatic disease [1]. Despite major advances in cancer immunotherapy, osteosarcoma remains a setting in which clinical benefit has been limited and inconsistent. Standard therapy relies on surgery and multi-agent chemotherapy [2], and multiple immunotherapy modalities have been evaluated with mixed results [3]. Immune checkpoint inhibitors targeting Programmed Cell Death Protein 1 (PD–1) and Programmed Death-Ligand 1 (PD-L1) have generally produced low response rates as monotherapy in osteosarcoma, with durable clinical benefit restricted to uncommon subsets [4]. Other approaches, including adoptive cellular therapies [5], cancer vaccines [6] and cytokine-based strategies [7] have shown promise in selected preclinical and early clinical settings but have not yet translated into broadly effective treatments. Collectively, these data suggest that osteosarcoma may require immunotherapy strategies that extend beyond T-cell checkpoint blockade alone and instead target dominant non-T-cell immune compartments within the tumor microenvironment. In osteosarcoma, tumor-associated macrophages (TAMs) are frequently abundant and can be immunosuppressive, yet retain the capacity to execute anti-tumor effector functions if appropriately reprogrammed [8].

Monoclonal antibodies (mAbs) targeting CD47 have emerged as a promising macrophage-based immunotherapy across multiple malignancies, including osteosarcoma [9–11]. CD47 functions as an innate immune checkpoint by binding Signal-Regulatory Protein Alpha (SIRPα) on TAMs, thereby delivering a “don’t eat me” signal that inhibits phagocytosis [12]. Anti-CD47 mAb therapy blocks the CD47-SIRPα interaction, thereby activating TAMs to eliminate osteosarcoma cells [13]. In preclinical osteosarcoma models, CD47 blockade significantly enhanced macrophage phagocytosis and inhibited tumor growth [11].

Prior preclinical investigations of CD47 blockade in osteosarcoma were performed in early-stage or low-volume disease models and did not systematically evaluate established tumors across a defined range of baseline volumes. For example, Xu et al. [11] initiated anti-CD47 treatment three days after intratibial implantation of KRIB osteosarcoma cells and reported a significant reduction in mean tibial tumor weight (430 mg versus 841 mg in IgG controls, p < 0.001) and decreased pulmonary metastasis incidence (13% versus 75%, p < 0.0001), without reporting baseline tumor volume or stratifying response by tumor size. Thus, whether increasing tumor burden directly limits macrophage activation and response to CD47 blockade remains unknown [14].

Emerging evidence from preclinical studies suggests that tumor size can influence the efficacy of cancer immunotherapies. Triplett et al. found that murine fibrosarcoma and colon carcinoma tumors under 50 mm^2^ responded robustly to anti-OX40 immunotherapy, with increased T-cell activation, whereas larger tumors (50 and 120 mm^2^) showed markedly reduced responses [15]. Similarly, Morrison et al. found that murine pancreatic tumors smaller than 50 mm^3^ demonstrated significantly enhanced responses to CD40 agonist and checkpoint blockade therapies, characterized by increased T-cell activation compared with tumors exceeding 50 mm^3^ [16]. Guisier et al. found that mice harboring smaller lung squamous cell carcinomas (~30 mm^3^) displayed a markedly enhanced response to PD–1 blockade, associated with increased infiltration of regulatory T-cells (Tregs) [17]. These studies provide quantitative evidence that tumor size can determine immunotherapy responsiveness. However, this size-dependent relationship has not been directly investigated for CD47 blockade in osteosarcoma, nor has it been linked to a macrophage-specific quantitative imaging biomarker. In addition, early-phase clinical trials of CD47-targeting antibodies in patients with advanced solid tumors evaluated treatment response using RECIST criteria but did not report outcomes stratified by baseline tumor size [18–20]. Therefore, whether tumor burden contributes to the limited objective responses observed in advanced disease remains unclear. A systematic study of tumor-size-dependent response in controlled preclinical osteosarcoma models is needed to address this gap.

Noninvasive imaging approaches offer a unique opportunity to assess how tumor size influences immunotherapy responses *in vivo*. We have developed and validated a clinically translatable biomarker for activated TAMs using ferumoxytol-enhanced magnetic resonance imaging (MRI) in osteosarcomas [21]. Ferumoxytol is an FDA-approved ultrasmall superparamagnetic iron oxide nanoparticle that is internalized by macrophages through phagocytosis. CD47 blockade enhances macrophage-mediated phagocytosis by disrupting the tumor cell “don’t eat me” signal, thereby promoting uptake of ferumoxytol by TAMs. As a result, TAMs with greater phagocytic activity accumulate and retain higher concentrations of ferumoxytol, leading to increased susceptibility-related signal loss and T2* shortening on MRI [22]. Accordingly, ferumoxytol-enhanced MRI can serve as a noninvasive imaging biomarker of intratumoral macrophage-mediated nanoparticle uptake, providing information on TAM abundance, spatial distribution, and phagocytic function [21]. Ferumoxytol-enhanced MRI measures nanoparticle uptake by macrophages, which serves as an indirect indicator of macrophage phagocytic activity following CD47 blockade. This imaging biomarker is most informative when combined with histological validation.

By combining ferumoxytol-enhanced MRI with CD47 mAb therapy, we monitored TAM activation and determined whether baseline tumor size affected immune response. To our knowledge, no prior study has investigated the impact of baseline tumor size on macrophage activation or imaging response to CD47 mAb therapy in osteosarcoma. We hypothesized that smaller tumors would exhibit greater TAM activation and higher ferumoxytol uptake compared with larger tumors following CD47 blockade. Therefore, the purpose of this study was to determine whether tumor size influences macrophage activation and molecular imaging response to CD47 mAb therapy.

## 2. Methods

### 2.1. Cell lines

Murine macrophages RAW 264.7, the murine K7M2, and human 143B osteosarcoma cell lines were obtained from the American Type Culture Collection (ATCC, Manassas, VA, USA). The cells were cultured in Dulbecco’s Modified Eagle’s Medium (DMEM) supplemented with 10% fetal calf serum, 100 μg/mL streptomycin, 100 U/mL penicillin, and 2 mM L-glutamine. All cell culture supplements were sourced from Gibco (Invitrogen, Carlsbad, CA, USA). Cell line authentication was performed using short tandem repeat (STR) profiling to confirm cell identity. All cell lines were routinely tested and confirmed to be free of mycoplasma contamination before use in experiments.

### 2.2. CCK–8 Cell Viability Assay

Cell viability following ferumoxytol treatment was assessed using the Cell Counting Kit–8 (CCK–8; GK10001, GLPBIO, USA). RAW 264.7 macrophages were seeded into 96-well plates at a density of 5 × 10 ^3^ cells per well and cultured overnight at 37°C in a humidified incubator containing 5% CO_2_. Cells were subsequently incubated with varying concentrations of ferumoxytol (25–200 μg/mL) for 24 h. Then, 10 μL of CCK–8 reagent was added to each well, and the plates were incubated for 1 h at 37 °C. Absorbance was measured at 450 nm using a microplate reader. Cell viability was normalized to untreated control cells and expressed as a percentage of the untreated control group.

### 2.3. Mouse models

Thirty four- to five-week-old female BALB/c mice (Jackson Laboratory, Bar Harbor, ME, USA) received an intratibial implantation of 1 × 10^6^ K7M2 tumor cells. Thirty female NOD SCID gamma (NSG) mice (Charles River Laboratories, Wilmington, MA, USA) received an intratibial implantation of 1 × 10^6^ 143B cells. Primary tumor growth was monitored daily by visual inspection and caliper measurements. A schematic summary of the study design is presented in Fig. 1. To determine the effect of CD47 mAb on primary osteosarcomas of different sizes, thirty animals with intratibial 143B osteosarcomas (group 1) and thirty animals with intratibial K7M2 osteosarcomas (group 2) were randomly assigned to three subgroups of ten mice each: small tumors with a tumor volume of ~ 30 mm^3^, medium-sized tumors with a tumor volume of ~ 500 mm^3^, and large tumors with a tumor volume of ~ 1200 mm^3^. The 500 mm^3^ cutoff was chosen to represent established, measurable tumors prior to development of necrosis and vascular insufficiency, while 1200 mm^3^ represents advanced tumors approaching humane endpoint criteria where perfusion and hypoxia are commonly altered. These thresholds were also selected to create two well-separated baseline states to maximize power for detecting size-dependent differences with feasible animal numbers.

Within each tumor size group, five animals were randomly assigned to one of two different treatments: phosphate-buffered saline (PBS) or anti-CD47 monoclonal antibody (mAb), delivered via intraperitoneal injection on days 1, 3, and 5 after a baseline MRI scan. Murine K7M2 tumors were treated with murine CD47 mAb (clone MIAP301, BioXcell, Lebanon, NH, USA), and human 143B tumors were treated with a human CD47 mAb (clone B6H12, BioXcell, Lebanon, NH, USA). All animal experiments were conducted in compliance with the Stanford University Administrative Panel on Laboratory Animal Care (Protocol 24965).

### 2.4. Ferumoxytol-enhanced MRI of small and large osteosarcomas

To evaluate the effect of tumor size on CD47 mAb-mediated macrophage activation and nanoparticle phagocytosis, all animals in groups 1 and 2 underwent MRI on a preclinical 7 T MRI scanner (Bruker Corporation, Billerica, MA, USA). All animals underwent baseline MRI (pretreatment pre-contrast) on day 1, followed by treatment with CD47 mAb or PBS on days 1, 3, and 5. Post-treatment pre-contrast MRI was performed on day 7, followed by infusion of ferumoxytol (30 mg Fe/kg body weight) and post-treatment post-contrast MRI on day 8. MRI scans were obtained with a T2-weighted fast spin echo (FSE) sequence with a repetition time (TR) of 2500 ms and an echo time (TE) of 33 ms as well as a multi-gradient echo (MGE) sequence for T2*-mapping with a flip angle of 50 °. The MGE sequence acquired 10 echo images with TEs from 3.5 ms to 48.5 ms (inter-echo time of 5 ms) and a TR of 800 ms. All images were obtained with a field of view of 2.56 cm × 2.56 cm, and an image matrix of 256 × 256. The tumor volume was measured on T2-FSE images using OsiriX version 8.0 (Pixmeo SARL, Bernex, Switzerland). The tumor MRI signal was quantified as T2* relaxation times, which were measured on T2*-maps using Paravision 7.1 software (Bruker Corp, Billerica, MA) as described [23]. To address whether size-dependent differences in tumor heterogeneity exist, baseline tumor heterogeneity was quantified prior to any therapeutic intervention on T2*-weighted MRI scans. For each tumor, the coefficient of variation (CV) of tumor T2* signal intensity was calculated as: CV (%) = [SD(T2*) / mean(T2*)] × 100 where SD and mean represent the standard deviation and average T2* signal intensity within the tumor region of interest, respectively. CV quantifies normalized signal dispersion, reflecting spatial variation in tumor biology. All image analyses were performed in a blinded manner to prevent observer bias.

### 2.5. Histopathological Evaluation

Directly after the final MRI scan, mice in groups 1 and 2 were euthanized. Tumors were excised and processed for histological analyses. Tumors were decalcified, fixed in 10% formalin, dehydrated, embedded in paraffin, and sectioned into 5 μm-thick slices. Tumor sections were stained with hematoxylin and eosin (H&E) or processed for iron detection with the Iron Stain Kit (HT20, Sigma-Aldrich Inc., MO, USA). For iron staining, sections were deparaffinized, rehydrated, and incubated with potassium ferrocyanide/hydrochloric acid for 20 min followed by DAB incubation (mouse- and rabbit-specific HRP/DAB micropolymer detection kit, ab236466, Abcam, Cambridge, UK) for 10 min. Sections were then counterstained with pararosaniline, dehydrated, and mounted.

Stitched whole-slide mosaics were acquired (NanoZoomer, Hamamatsu Photonics, Shizuoka, Japan) and analyzed using QuPath software (version 0.5.1) [24]. Tissue regions were segmented using average channel thresholding (Gaussian prefilter σ = 2, threshold = 215–225), and iron-positive areas were quantified as percentage area using a pixel classifier (DAB channel, σ = 2, threshold = 0.1–0.4).

To correlate tumor ferumoxytol MRI enhancement with TAMs on histology, double-immunofluorescence staining was performed for F4/80 with either CD80 (M1-TAM marker) or CD206 (M2-TAM marker), as described [25]. Paraffin sections were deparaffinized, fixed in 4% paraformaldehyde in PBS (pH 7.4), permeabilized in 0.25% Triton X–100 (PBST) and blocked in 1% BSA in PBST. After blocking, sections were incubated overnight at 4 °C with rat monoclonal anti-F4/80 (1:200, ab6640, Abcam, Cambridge, UK) combined with either rabbit polyclonal anti-CD80 (1:200, ab254579, Abcam) or anti-CD206 (1:200, ab64693, Abcam). Sections were then incubated with secondary antibodies: Alexa Fluor 488 (A–11006, Invitrogen, Carlsbad, CA, USA) and Alexa Fluor 647 (A–21245, Invitrogen, Carlsbad, CA, USA). Finally, slides were mounted with DAPI-containing mounting medium (00–4959–52, Invitrogen, Thermo Fisher Scientific, Waltham, MA, USA).

Immunofluorescence images were captured using a Leica TCS SP8 confocal microscope (DMI6000; Leica Microsystems, Wetzlar, Germany) at × 20 magnification. Image analyses were performed using ImageJ/Fiji and MATLAB (MathWorks, Natick, MA, USA) [26]. Mean fluorescence intensity (MFI) values (DAPI, F4/80, CD80/CD206) were measured in three randomly selected fields of view (FOVs) per slide, normalized to DAPI, and averaged per group [27]. The M1/M2 ratio was calculated as the ratio of CD80 MFI to CD206 MFI after normalization of each marker to DAPI MFI. All image analyses were performed in a blinded manner to prevent observer bias. The relative expression of CD80 and CD206 to F4/80 was assessed according to tumor size and treatment group.

### 2.6. Statistical Analysis

Power calculations were informed by prior preclinical MRI studies in osteosarcoma-bearing mice and indicated that a minimum of five mice per group (small, medium, and large tumors) were needed to detect significant differences in imaging biomarkers. Assuming all CD47 mAb-treated mice responded, and all controls did not, this sample size provided 90% power (two-sided α = 0.05) to detect differences of ≥ 1.5 standard deviations. For correlation analyses between tumor size and nanoparticle enhancement or TAM signal, all fifteen mice across the three tumor-size groups were included, providing 90% power to detect correlations as low as r = 0.65.

MRI and histology data are presented as mean ± standard deviation (SD). Changes in post-treatment tumor T2* relaxation times before and after ferumoxytol infusion were calculated as follows: ΔT2* (%) = [T2* post-treatment pre-contrast – T2* post-treatment post-contrast] / T2* post-treatment pre-contrast * 100.

Comparisons between the CD47 mAb treatment group and the control group (PBS) were performed across tumors of varying sizes (small, medium, and large) to assess effects on T2* relaxation times, intratumoral heterogeneity, Prussian blue-positive percentage area, TAM markers (F4/80, CD80, CD206) and M1/M2 ratio. Multiple regression models, including an interaction term between treatment and tumor size, were used to evaluate both differences between treatments within each tumor size (PBS versus CD47 mAb) and differences among tumor sizes (small, medium, and large) within each treatment group. Model-based marginal effects were used for these comparisons, and p-values from the corresponding regression tests were used to assess statistical significance (p < 0.05 = *, p < 0.01 = ^**^, p < 0.001 = ^***^).

To further investigate the relationship between the treatment effect of ΔT2* and tumor size, additional analyses were conducted treating tumor size as a continuous variable. We correlated tumor size with MRI biomarkers (T2* relaxation time, ΔT2*, intratumoral heterogeneity) and histological markers (F4/80, CD80, CD206, M1/M2 ratio) within the CD47 mAb treatment group and the PBS control group. Exponential (non-linear) regression was used to evaluate the relationship between primary tumor size and tumor T2* relaxation times on ferumoxytol-MRI, with goodness-of-fit assessed using the coefficient of determination (R^2^). Linear regression was used to assess correlations between tumor size and histological TAM markers (F4/80, CD80, CD206, M1/M2 ratio) and Prussian blue–positive area (%), with the strength of the associations quantified by the Pearson correlation coefficient (r).

Two Poisson regression models were fit for both PBS and CD47 treatment groups. The expectation of each observation from the model can be expressed by

$$E\left(Y_{j}|X_{j}\right)=exp\left(a_{j}+b_{j}X_{j}\right)$$

where $x_{j}$ is the tumor size and the $y_{j}$ is the observed change in ΔT2* time, j = 1 for the CD47 group, and j = 2 for the PBS group. With estimated $\left(a_{j},b_{j}\right)$, the between group difference of the ΔT2* is

$$D(x)=expexp\left(a_{1}+b_{1}x_{1}\right)-expexp\left(a_{2}+b_{2}x_{2}\right)$$

The derivative of this between group difference is given by

$$\Delta (x)=b_{1}expexp\left(a_{1}+b_{1}x_{1}\right)-b_{2}expexp\left(a_{2}+b_{2}x_{2}\right),\;x\in \left[0,b_{0}\right]$$

The goal of this analysis is to show that the treatment effect is a decreasing function of size, achieved when the derivative is Δ(x)<0 for specific tumor sizes. Estimates for Δ(x) were obtained and then resampled to construct a confidence band for Δ(x) such that

$$P\left(L(x)<\Delta (x)<U(x),x\in \left[0,b_{0}\right]\right)=0.95$$

The between group difference is then said to be a strictly decreasing function of tumor sizes up to $b_{0}$.

Analyses were performed using Stata 18 (StataCorp LLC, College Station, TX) and R version 4.4.1.

## 3. Results

### 3.1. Effects of Ferumoxytol on RAW 264.7 Macrophages

RAW 264.7 macrophages were treated with ferumoxytol (25–200 μg/mL) for 24 h, and cell viability was assessed using the CCK–8 assay. Following background correction and normalization to untreated controls, no reduction in cell viability was observed at any ferumoxytol concentration tested. Viability remained above control levels across all treatment groups, ranging from 111.7 ± 10.8% to 128.3 ± 17.5% (Figure S1). These findings indicate that ferumoxytol does not induce cytotoxicity in RAW 264.7 macrophages at concentrations up to 200 μg/mL following 24 h exposure.

### 3.2. Ferumoxytol-enhanced MRI of small and large osteosarcomas

At baseline (pretreatment, pre-contrast), all osteosarcomas presented as intratibial tumors with extratibial soft tissue components that were hyperintense on T2-weighted MRI relative to skeletal muscle. Following treatment with either PBS or CD47 mAb, all tumors maintained hyperintensity on post-treatment pre-contrast scans.

#### Small 143B tumors:

On ferumoxytol-enhanced MRI, CD47 mAb-treated tumors demonstrated stronger hypointense enhancement compared with PBS-treated tumors, indicating TAM activation (Fig. 2A). CD47 mAb-treated tumors demonstrated significantly shorter post-contrast T2* relaxation times compared with PBS-treated tumors (p < 0.001, Fig. 2B) and significantly higher ΔT2* values compared with PBS-treated small tumors (p < 0.001, Fig. 2C).

#### Large 143B tumors:

In contrast, large tumors exhibited only modest hypointense signal enhancement after CD47 mAb and PBS treatment, suggesting attenuated TAM activation (Figs. 2D and 2E). CD47 mAb-treated tumors and PBS-treated tumors demonstrated no significant difference in ΔT2* values (p = 0.413, Fig. 2F).

#### Comparison of small and large 143B tumors:

CD47 mAb-treated small tumors showed significantly greater ferumoxytol enhancement (ΔT2* = 41.29% ± 1.89%) than large tumors (ΔT2* = 0.98% ± 0.54%; p < 0.001), indicating stronger TAM activation in smaller tumors. Furthermore, an inverse correlation was noted between tumor size and ferumoxytol-induced ΔT2* enhancement in CD47 mAb-treated tumors (R^2^ = 0.91, p < 0.001, Fig. 2G). The difference in ΔT2* enhancement between PBS and CD47 mAb-treated tumors decreased with increasing tumor size, suggesting that the therapeutic efficacy of CD47 mAb decreased as tumor burden increased.

To provide a more granular dose-response assessment, intermediate volume strata are described in detail in Supplementary File 1 and Figure S2.

#### Second tumor model:

To validate our findings, we repeated the experiment in murine K7M2 osteosarcomas (Supplementary File 1 and Figure S3). Consistent with the results observed in the human 143B tumor model, CD47 mAb-treated small K7M2 tumors exhibited a significant inverse correlation between tumor size and ferumoxytol-induced ΔT2* enhancement (R^2^ = 0.96, p < 0.001, Fig. 2H). As tumor size increased, the ΔT2* enhancement gap between PBS- and CD47 mAb-treated tumors narrowed, indicating that CD47 mAb efficacy declined with greater tumor burden. In nonlinear modeling, the estimated treatment-minus-control difference in ΔT2* decreased with increasing tumor size from 0 to 1500 mm^3^. For tumor sizes > 1500 mm^3^, the trend was no longer statistically significant (Figure S4).

#### Effects of intratumoral heterogeneity on CD47 mAb efficacy:

Prior to treatment initiation, large 143B tumors exhibited significantly greater intratumoral heterogeneity compared to small tumors (mean difference: 14.26%, 95% CI: 9.78–19.14, p < 0.001, Fig. 3). Baseline tumor heterogeneity did not differ between treatment and control groups in either small (p = 0.73) or large (p = 0.92) tumors.

### 3.3. Histopathological analysis of small and large osteosarcomas

Histology paralleled the MRI findings. CD47 mAb-treated 143B human osteosarcomas showed stronger Prussian blue staining than PBS controls (Fig. 4A), indicating increased ferumoxytol deposition via TAM-mediated phagocytosis. Among treated lesions, small tumors showed significantly greater staining than large tumors (Fig. 4B; p < 0.001), consistent with the stronger nanoparticle signal and larger ΔT2* seen on MRI in small tumors. Tumor size was significantly associated with Prussian blue staining in CD47 mAb-treated tumors (Fig. 4C; r = −0.70; p = 0.004), with larger tumors exhibiting less iron uptake. Moreover, the staining difference between PBS and CD47 mAb groups diminished as tumors enlarged, suggesting reduced CD47 mAb efficacy with increasing tumor burden.

Immunofluorescence analyses revealed increased staining for F4/80^+^ and CD80^+^ TAMs in small tumors treated with CD47 mAb compared with PBS-treated controls (Fig. 5A). In contrast, in large tumors, CD47 mAb did not increase F4/80^+^CD80^+^ or F4/80^+^CD206^+^ signal compared with PBS (Fig. 5B, p = 0.97 and p = 0.91, respectively), indicating limited TAM activation at high tumor burden. Quantification confirmed significantly greater F4/80^+^CD80^+^ staining in small CD47 mAb-treated tumors than in PBS-treated tumors (Fig. 5C, p < 0.001), suggesting enhanced M1 macrophage activation.

In the CD47 mAb-treated group, F4/80^+^CD80^+^ staining was significantly higher in small compared with large tumors (p = 0.006, Fig. 5C). Tumor size showed a significant inverse correlation with F4/80^+^CD80^+^ staining (Fig. 5D, r = −0.73, p = 0.002). The treatment-control difference in F4/80^+^CD80^+^intensity narrowed as tumors enlarged, indicating that CD47 mAb–induced TAM activation declined with increasing tumor burden (Figure S5).

The K7M2 osteosarcoma model confirmed these findings, showing a similar inverse correlation between tumor size and F4/80^+^CD80^+^ staining after CD47 mAb treatment (Figure S6).

To assess the impact of CD47 blockade on macrophage polarization, the M1/M2 ratio was quantified in tumors stratified by size. In both 143B and K7M2 tumor models, CD47 mAb treatment significantly increased the M1/M2 ratio in small tumors compared with PBS controls (143B, p = 0.04; K7M2, p = 0.03) (Figure S7). No significant differences were observed in medium or large tumors in either model (all p > 0.05). These results demonstrate that CD47 blockade promotes macrophage polarization toward an M1-like phenotype specifically in small tumors, with no detectable effect in larger tumors across both models.

## 4. Discussion

We found that MRI and histologic responses to CD47 mAb differed by baseline tumor size, with small tumors showing significantly greater decreases in T2* relaxation times and significantly higher F4/80^+^CD80^+^ macrophage signal than large tumors. These findings demonstrate that baseline tumor size is an independent determinant of macrophage activation and molecular imaging response to CD47 mAb therapy in osteosarcoma.

Prior work established CD47 as a therapeutic target and demonstrated macrophage-mediated tumor suppression in osteosarcoma models [11]. The present study extends these findings by identifying tumor size as a critical determinant of CD47 mAb efficacy. Importantly, earlier osteosarcoma studies did not stratify tumors by predefined baseline volumes and primarily evaluated early-stage tumors. In contrast, our study directly isolates tumor volume as an independent variable and demonstrates a size-dependent reduction in macrophage activation under otherwise identical treatment conditions. Specifically, we show that small tumors exhibit significantly greater macrophage activation and ferumoxytol imaging response compared with large tumors. These findings reveal a fundamental biological limitation of CD47 immunotherapy that was not addressed in earlier studies. Furthermore, these findings substantially extend our previous investigations demonstrating macrophage-dependent antitumor effects following CD47 blockade [25]. In contrast to earlier studies, which did not stratify tumors by size or examine imaging biomarkers of macrophage activation, the current study directly addresses a critical translational question: whether tumor burden influences innate immune checkpoint efficacy. Early-phase CD47 clinical trials did not report responses stratified by baseline tumor size, and therefore, a direct clinical correlation between tumor burden and treatment efficacy has not yet been established. Our data provide preclinical evidence that increasing tumor volume attenuates macrophage activation following CD47 blockade.

Although larger tumor burden has been broadly associated with reduced responsiveness to immunotherapy, this concept has not previously been demonstrated for CD47 blockade in osteosarcoma, nor mechanistically linked to macrophage-specific molecular imaging biomarkers.

Our data provide direct evidence that macrophage activation in response to CD47 mAb therapy is significantly impaired in larger tumors. This observation suggests that failure to adequately activate TAMs in bulky tumors may represent a previously unrecognized mechanism of therapeutic resistance. To further elucidate the underlying mechanisms, we examined baseline intratumoral heterogeneity as a potential contributor to size-dependent treatment resistance. Our findings reveal that intratumoral heterogeneity at baseline contributes to impaired macrophage activation and reduced CD47 mAb efficacy. Large tumors exhibited higher baseline heterogeneity compared to small tumors. Intratumoral heterogeneity likely reflects spatial barriers to antibody penetration and immune cell infiltration in large tumors, creating regions with differential treatment exposure and macrophage activation. These findings demonstrate that tumor burden at treatment initiation is a critical determinant of CD47 mAb efficacy.

Previous studies have highlighted CD47 as a promising immune checkpoint target, particularly for tumors of mesenchymal origin such as osteosarcoma, which are often resistant to T-cell-based immunotherapies [28,29]. Our data add a critical dimension to this work by showing that the therapeutic efficacy of CD47 mAb is significantly attenuated in large solid tumors, underscoring the need to consider tumor burden when stratifying patients for immunotherapy treatment.

Our findings are consistent with reports from other solid tumor models, suggesting that the immunosuppressive tumor microenvironment (TME) becomes increasingly hostile to innate immune effectors as tumors progress [30,31]. Larger tumors are associated with higher levels of hypoxia, necrosis, and immunosuppressive cytokines such as TGF-β and IL-10, which may inhibit macrophage infiltration and polarization toward a pro-inflammatory (M1-like) phenotype [32–34]. Indeed, we observed reduced expression of both CD80^+^ and CD206^+^ TAM markers in large tumors regardless of treatment, suggesting a general suppression or exclusion of macrophage activity in advanced osteosarcomas.

Although tumor size has been implicated in shaping antibody efficacy, our study is the first to demonstrate that tumor size is a critical determinant of response to macrophage-directed CD47 blockade in osteosarcoma. Hopkins et al. [35] examined 1461 patients with non-small cell lung cancer (NSCLC) treated with atezolizumab and found that smaller baseline tumor size (< 6.4 cm) was associated with significantly longer progression-free survival (PFS) (HR 1.21 [95% CI 1.07–1.38], p = 0.003) and overall survival (OS) (HR 1.64 [95% CI 1.41–1.91], p < 0.001) compared with larger tumors (> 6.4 cm). Katsurada et al. [39] evaluated the impact of baseline tumor size in 58 patients with NSCLC and found that smaller tumors (≤ 10.1 cm) were associated with longer PFS (median PFS 6.39 months) compared with larger tumors (> 10.1 cm; median PFS 2.07 months). Uehara et al. [38] confirmed this pattern in 159 patients with NSCLC, reporting better PFS in smaller tumors (≤ 14.7 cm) compared with those with larger tumors (> 14.7 cm). In patients with melanoma treated with pembrolizumab, Joseph et al. [36] found significantly higher objective response rates (44% versus 23%; p < 0.001) and OS (HR, 0.38; p < 0.001) in those with smaller tumors (baseline tumor size ≤ 10.2 cm) compared with larger tumors (baseline tumor size > 10.2 cm). Similarly, among 25 patients with platinum-refractory recurrent and metastatic head and neck squamous cell cancer, Suzuki et al. [37] observed longer PFS and OS in those with tumors < 31 mm compared with those with ≥ 31 mm. While these studies evaluated predominantly T-cell-directed checkpoint inhibitors, they provide quantitative evidence across multiple tumor types that increasing baseline tumor size is associated with diminished immunotherapy efficacy. Our study investigated this size-response relationship for macrophage-directed CD47 blockade and linked it to a measurable imaging biomarker (ΔT2*). To achieve this, we used ferumoxytol-enhanced MRI as a clinically translatable quantitative biomarker for TAM responses in osteosarcomas. Other investigators have used ferumoxytol-MRI to estimate the transendothelial delivery of macromolecular drugs to tumor tissue. Ramanathan et al. [40] used ferumoxytol-enhanced MRI to predict tumor delivery and response to nanoliposomal irinotecan in patients with a wide variety of metastatic solid tumors. These findings have direct clinical implications. Early-phase clinical trials of CD47 mAbs in patients with advanced solid tumors demonstrated acceptable safety but limited objective responses [19,20]. For example, Zeidan et al. [19] reported a phase I study of CC–90002 monotherapy in 24 patients with relapsed/refractory acute myeloid leukemia (AML) and 4 patients with high-risk myelodysplastic syndromes (MDS). Despite high disease burden (median bone marrow blast percentage 40.6%), no objective responses were observed (ORR 0%). Patnaik et al. [20]reported a phase I study of SRF231 monotherapy in 37 patients with advanced solid tumors evaluable by RECIST v1.1, showing no complete or partial responses (ORR 0%); however, the potential impact of baseline tumor size on treatment response was not assessed. Neither study reported baseline tumor size nor analyzed response as a function of tumor burden. Thus, it remains unknown whether tumor burden contributed to the limited objective responses observed. While clinical trials evaluated tumor size reduction by RECIST criteria, our study investigated macrophage activation using ΔT2* relaxation time, a mechanistic biomarker of response. Our data demonstrate that tumors measuring approximately 30 mm^3^ exhibited a significant increase in ΔT2* (ferumoxytol enhancement) and M1 macrophage activation, whereas tumors measuring approximately 1200 mm^3^ did not show a significant ΔT2* difference relative to controls. These findings suggest that tumor size alone can constrain CD47-mediated macrophage activation and should be prospectively evaluated as a biological variable in future translational studies.

The ferumoxytol dose was selected to achieve adequate tumor contrast-to-noise ratio for quantitative T2* mapping in mice, consistent with prior preclinical ferumoxytol MRI studies [41]. Nanoparticle doses in mice are typically higher compared with humans due to the shorter half-life and faster metabolism in rodents. Ferumoxytol has been used clinically for MRI at lower iron doses. Additional hepatic uptake is expected and does not confound tumor T2*quantification outside the liver in mice [42] or humans [43]. Clinical translation will require optimization of ferumoxytol dose and imaging time points.

Ferumoxytol labels both M1- and M2-polarized TAMs. Other investigators have developed more specific nanoparticles. For example, Zhou et al. [44] reported that sulfated-dextran-coated iron oxide nanoparticles (SDIO) could specifically label M2-polarized macrophages in vitro and in vivo in a mouse model of breast cancer. Li et al. [45] reported that M2pep-uIONP selectively targeted M2 macrophages. While SDIO and M2pep-uIONP are not currently approved for clinical use, ferumoxytol is FDA-approved as an iron supplement and can be used off-label as an imaging agent in patients [21,46,47].

These findings highlight baseline tumor burden as a key determinant of macrophage activation and underscore several important directions for future investigation. These results from xenograft and syngeneic mouse models require validation in prospective clinical cohorts treated with anti-CD47/SIRPα therapies. In addition, tumor ΔT2* and Prussian blue staining represent indirect readouts of TAM activation and may be influenced by tumor perfusion, hypoxia, and necrosis; integrating complementary measures such as perfusion imaging, CD31 staining, and quantitative necrosis assessment would help isolate macrophage-driven effects. Additionally, MRI and histology provide complementary but distinct information. While MRI quantifies whole-tumor average ΔT2*, histological analysis detects focal increases in TAM staining and iron with higher sensitivity, particularly when ferumoxytol distribution is heterogeneous. Section-level immunofluorescence revealed regional differences in macrophage phenotype not captured by global imaging metrics. We addressed this by analyzing spatial heterogeneity within MRI regions of interest to better correlate imaging and histological findings. T2* values can be influenced by tumor hemorrhage, endogenous iron, calcification, and necrosis. To account for these potential confounders, we standardized imaging parameters, applied consistent ROI selection criteria, and excluded nonviable regions. Correlation with histological iron staining and macrophage markers confirmed that observed T2* changes predominantly reflect ferumoxytol accumulation in TAM-rich regions. The current analyses were primarily size-stratified and cross-sectional, without longitudinal endpoints. Finally, clinical CD47 trials have not yet incorporated baseline tumor-size stratification or macrophage-specific imaging biomarkers, limiting direct translational comparison. Prospective clinical studies incorporating tumor-volume quantification and macrophage imaging readouts will be necessary to validate whether the size-dependent limitation observed here extends to human osteosarcoma.

## 5. Conclusion

In conclusion, our data reveal that the immunotherapeutic efficacy of CD47 mAb in osteosarcoma is significantly constrained by tumor size. These findings provide new mechanistic insights into the limitations of macrophage-targeted therapies in bulky disease. Specifically, tumors with low baseline volume demonstrated a significant increase in ΔT2* and M1 macrophage activation following CD47 blockade, whereas larger tumors did not exhibit a significant imaging or immunologic response. Together, these results establish baseline tumor size as a critical biological variable influencing macrophage activation and imaging response to CD47 mAb therapy. Prospective clinical studies should incorporate tumor-volume stratification to determine whether this size-dependent limitation extends to human osteosarcoma.

## Supplementary Material

1

2

## Figures and Tables

**Fig. 1.**
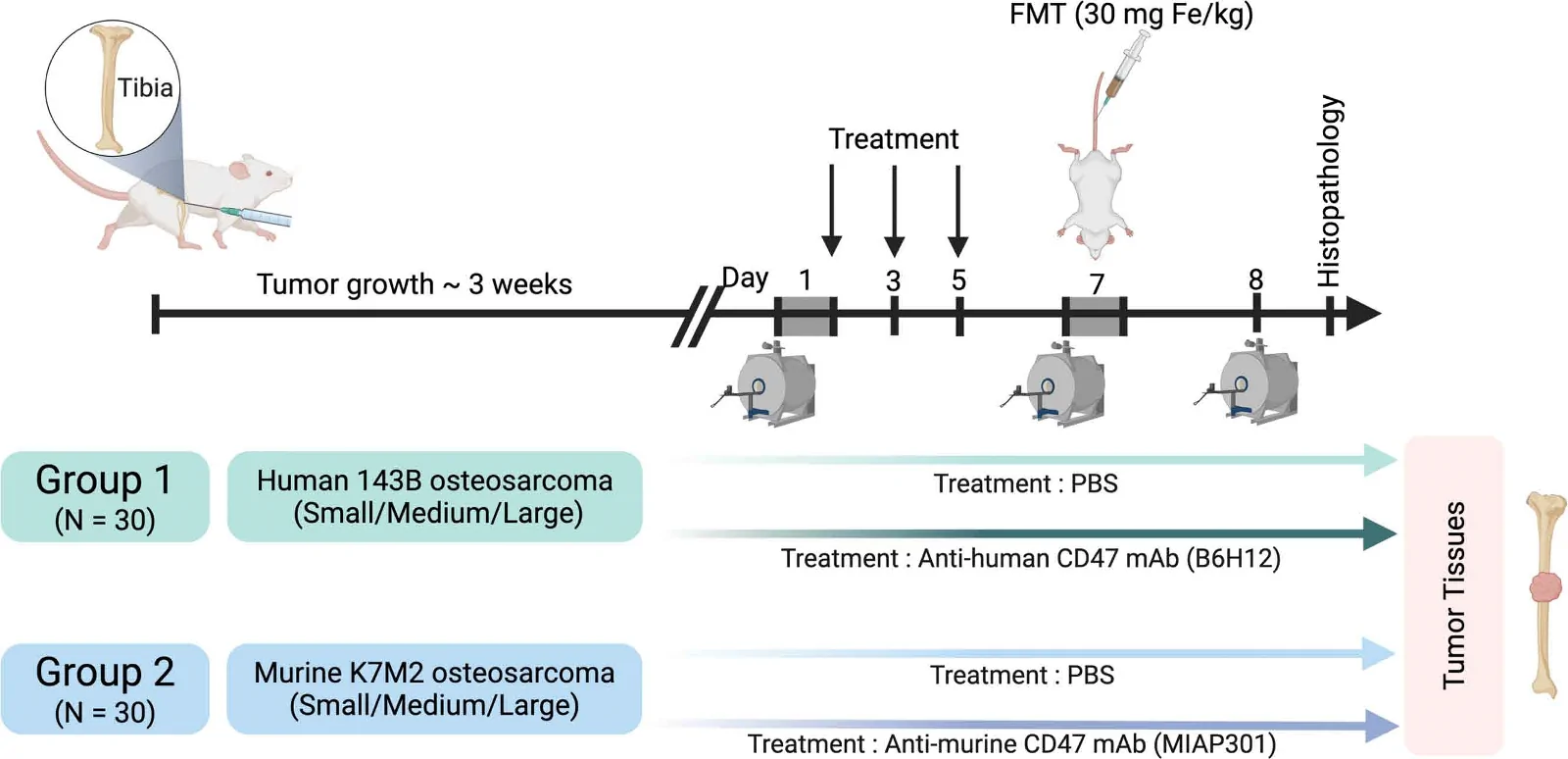
Experimental study design. Human 143B or murine K7M2 osteosarcoma cells were orthotopically engrafted into the tibia of NSG or BALB/c mice, respectively. Within each tumor group, mice were randomly assigned to treatment with CD47 monoclonal antibody (mAb, 10 mg/kg, n = 15) or phosphate-buffered saline (PBS, n = 15) on days 1, 3, and 5, followed by magnetic resonance imaging (MRI) before (day 7) and 24 h after (day 8) intravenous administration of ferumoxytol (FMT). After the final MRI, tumor specimens were harvested for histological examination.

**Fig. 2.**
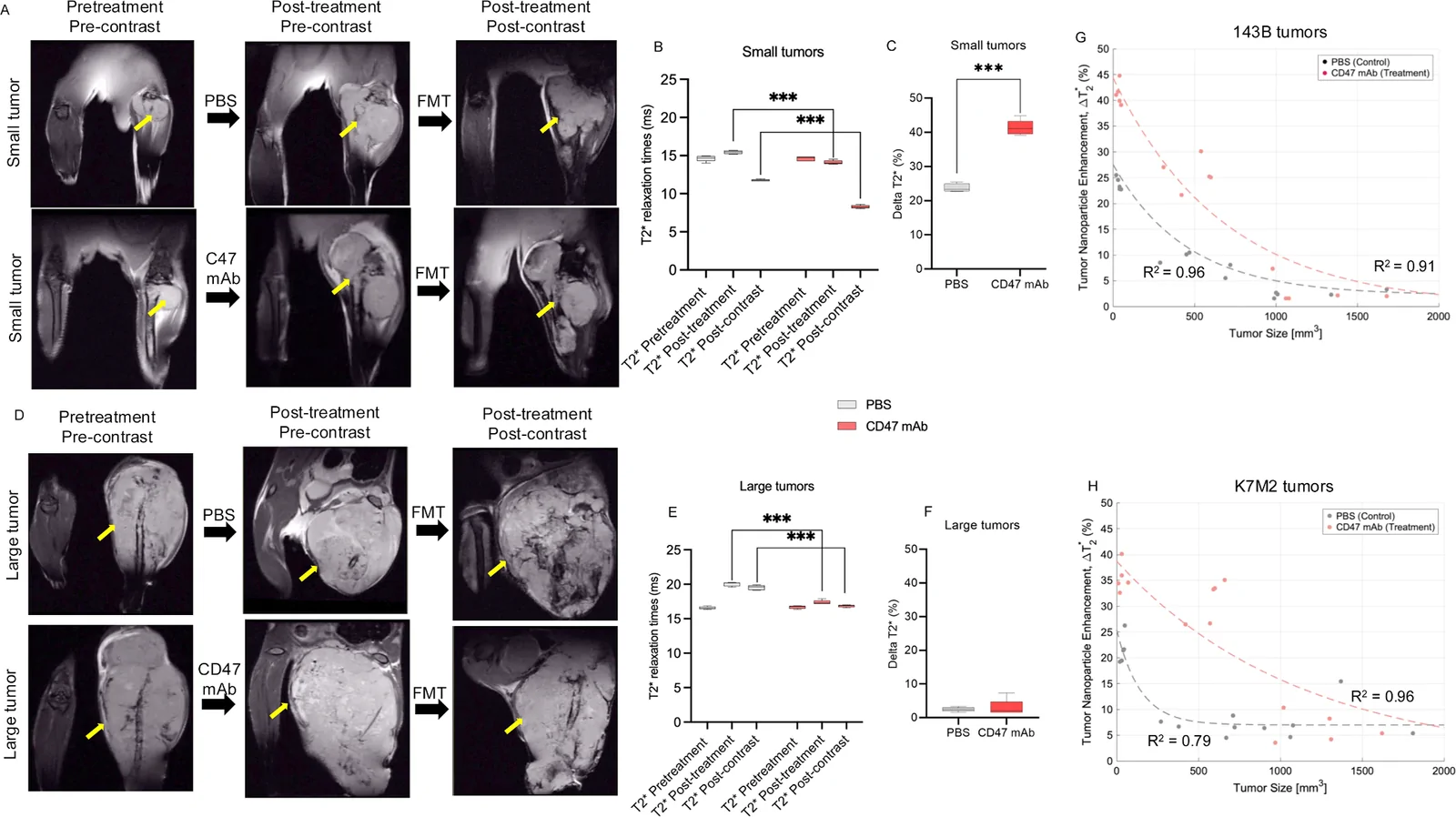
MRI of small and large 143B human osteosarcomas before and after treatment with PBS or CD47 mAb. (A) Representative coronal T2-weighted MRI of small 143B tumors at baseline (pretreatment, pre-contrast), after CD47 mAb or PBS treatment (post-treatment, pre-contrast), and 24 h after intravenous ferumoxytol injection (post-treatment, post-contrast). (B) Corresponding tumor T2* relaxation times (ms) of small tumors at baseline, post-treatment pre-contrast, and post-treatment post-contrast. (C) ΔT2* (percentage change in T2* relaxation time before and after ferumoxytol) of small tumors treated with CD47 mAb or PBS. (D) Representative coronal T2-weighted MRI of large 143B tumors at baseline (pretreatment, pre-contrast), after CD47 mAb or PBS treatment (post-treatment, pre-contrast), and 24 h after intravenous ferumoxytol injection (post-treatment, post-contrast). (E) Corresponding tumor T2* relaxation times (ms) of large tumors at baseline, post-treatment pre-contrast, and post-treatment post-contrast. (F) ΔT2* (percentage change) of large tumors treated with CD47 mAb or PBS. (G) Scatter plot of ΔT2* versus tumor size for PBS-treated (gray dots) and CD47 mAb-treated (pink dots) 143B tumors. Exponential regression yielded R^2^ = 0.96 for PBS and R^2^ = 0.91 for the CD47 mAb. (H) Scatter plot of the ferumoxytol tumor enhancement (ΔT2*) as a function of the primary tumor size for the PBS-treated (gray dots) and CD47 mAb-treated (pink dots) K7M2 tumors. Exponential regression yielded R^2^ = 0.79 for PBS and R^2^ = 0.96 for CD47 mAb. Data represent mean ± SD from five tumors per group. Only statistically significant comparisons are shown (*** p < 0.001).

**Fig. 3.**
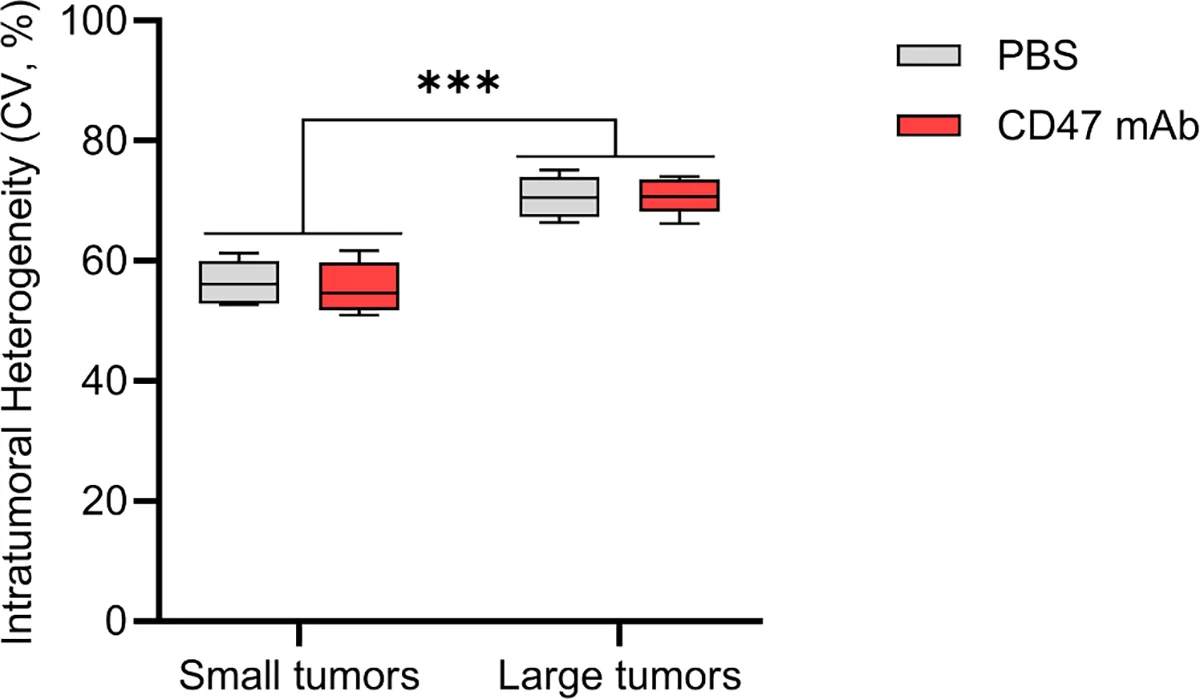
Baseline intratumoral heterogeneity in small and large 143B osteosarcoma tumors prior to PBS or CD47 mAb treatment. Intratumoral heterogeneity was quantified as the coefficient of variation (CV, %) of voxel-wise T2* relaxation values within tumor regions of interest using quantitative MRI. Only statistically significant comparisons are shown (*** p < 0.001).

**Fig. 4.**
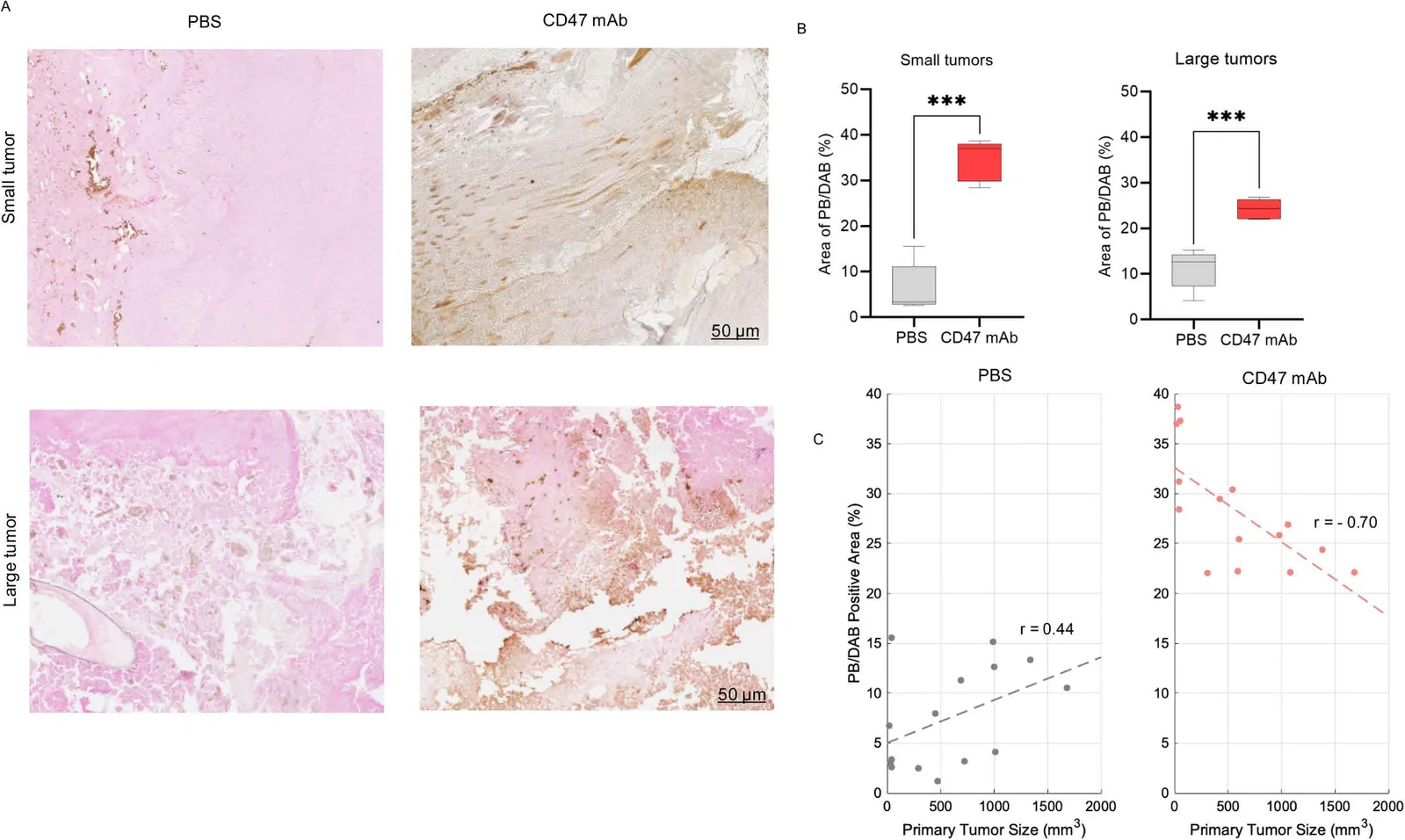
Prussian blue/DAB staining of human 143B osteosarcomas following PBS or CD47 mAb therapy. (A) Representative Prussian blue/DAB images of small and large 143B tumors after PBS or CD47 mAb therapy (20 × magnification). (B) Percentage area of Prussian blue/DAB-positive staining in small and large 143B tumors after PBS or CD47 mAb treatment. Data represent mean ± SD of five tumors per group. Only statistically significant comparisons are shown (***p < 0.001). (C) Scatter plot of percentage area of Prussian blue/DAB-positive staining as a function of tumor size for the PBS-treated (gray dots) and CD47 mAb-treated (pink dots) tumors. Linear regression yielded r = 0.44 for PBS and r = − 0.70 for CD47 mAb.

**Fig. 5.**
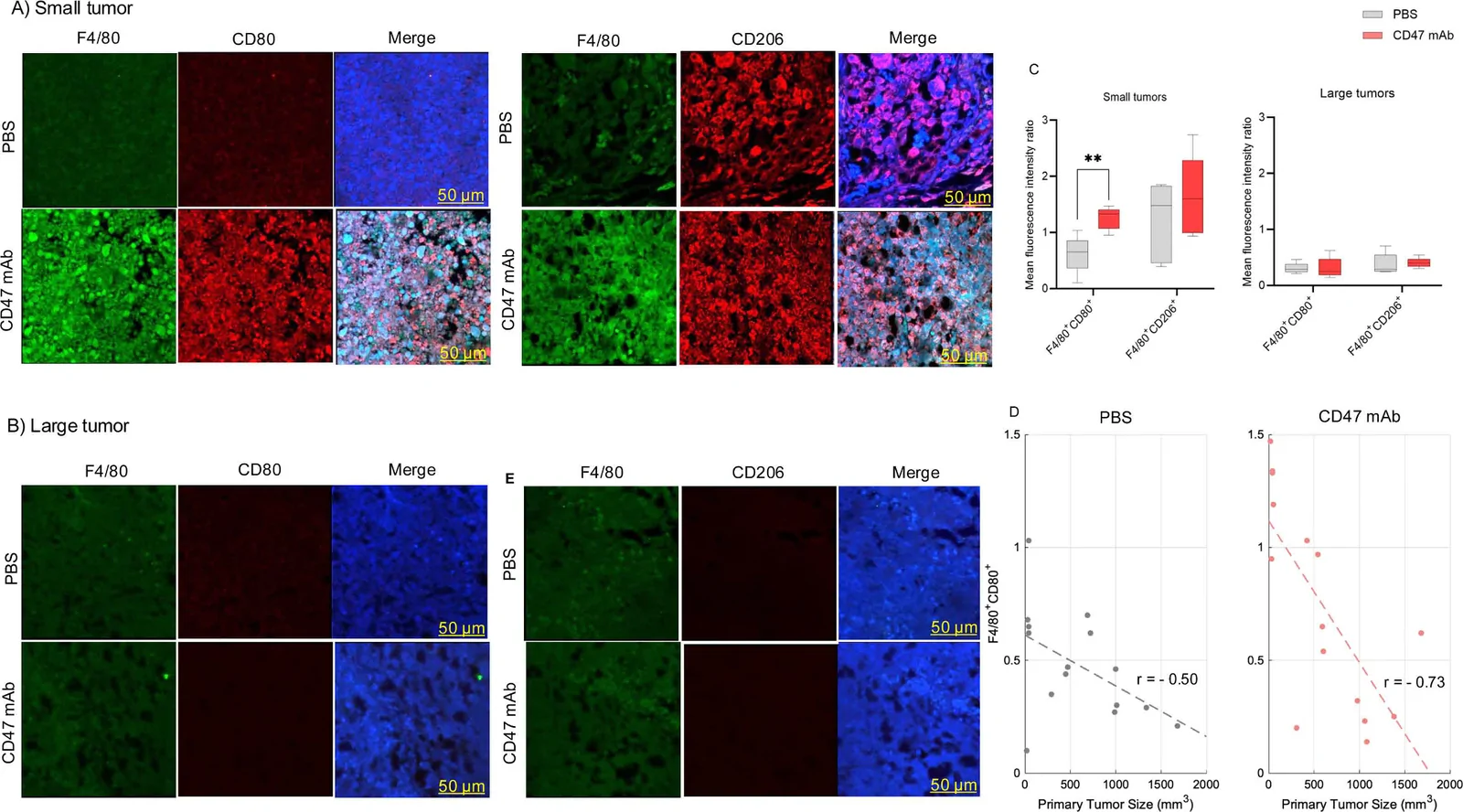
Histopathological analysis of tumor-associated macrophages (TAMs) in human 143B osteosarcomas after PBS or CD47 mAb treatment. (A) Representative immunofluorescence confocal micrographs (20 ×) of a small tumor treated with PBS or CD47 mAb, stained with F4/80^+^, CD80^+^ (M1), and CD206^+^ (M2) TAM markers. (B) Representative immunofluorescence confocal micrographs (20 ×) of a large tumor treated with PBS or CD47 mAb, stained for F4/80^+^, CD80^+^ (M1), and CD206^+^ (M2) TAM markers. (C) Mean fluorescence intensity of F4/80^+^CD80^+^ and F4/80^+^CD206^+^ TAMs in small and large tumors treated with PBS or CD47 mAb. Data represent mean ± SD from five tumors per group. Only statistically significant comparisons are shown (** p < 0.01). (D) Scatter plot of F4/80^+^CD80^+^ TAMs as a function of tumor size for the PBS-treated (gray dots) and CD47-mAb treated (pink dots) tumors. Linear regression yielding r = − 0.50 for PBS and r = − 0.73 for CD47 mAb.

## Data Availability

Data will be made available on request.

## Appendix A. Supporting information

Supplementary data associated with this article can be found in the online version at doi:10.1016/j.biopha.2026.119846.
