# Supplementary material for: Tumor size limits macrophage activation and imaging response to CD47 blockade in osteosarcoma
Source: Biomed Pharmacother. Author manuscript; Available in PMC 2026 Oct 28. (PMC13714959; doi:10.1016/j.biopha.2026.119846)
Supplement: Supplementary file 1 [file NIHMS2211656-supplement-2.pdf]

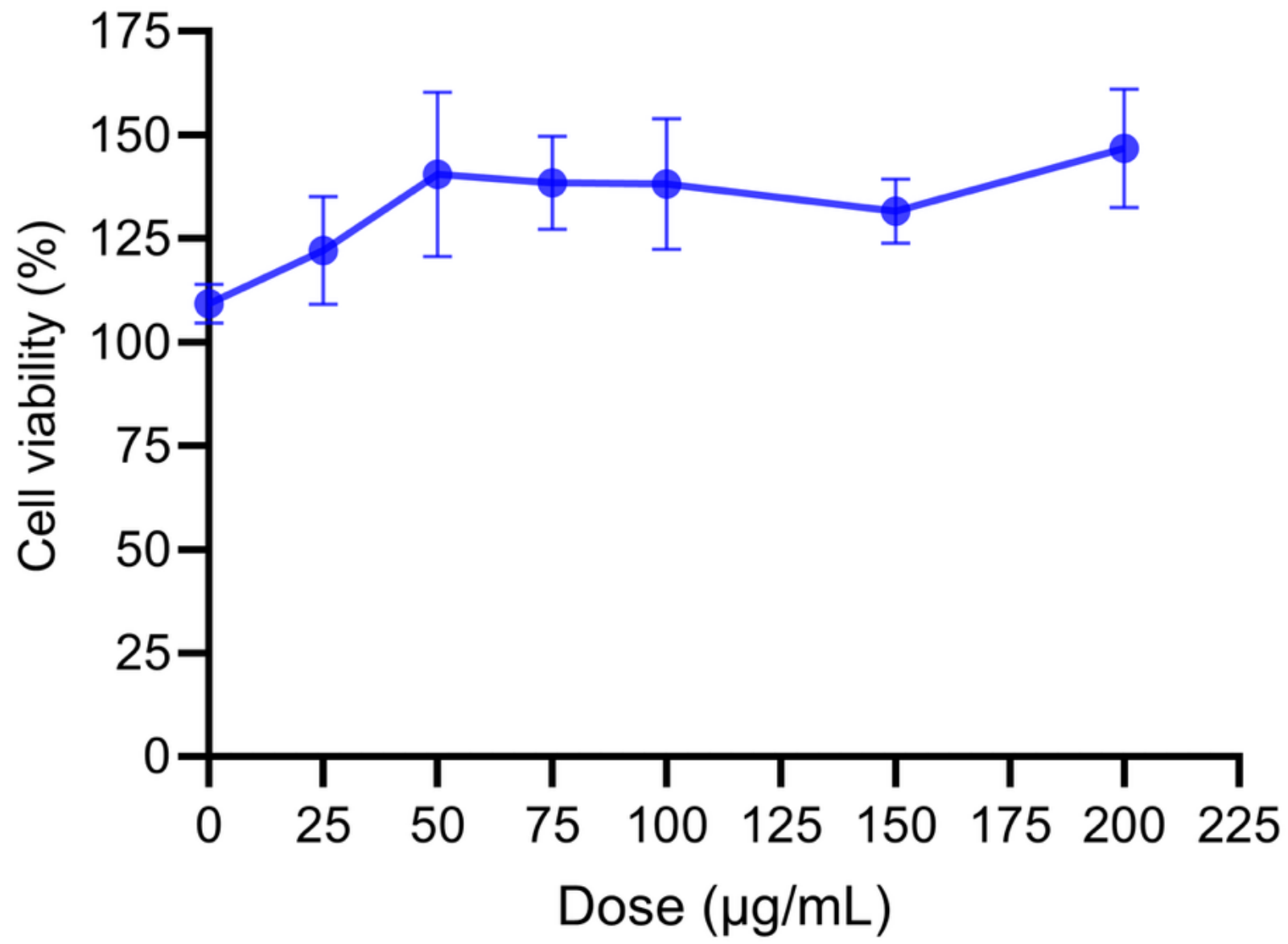

Cell viability (%)
175
150
125
100
75
50
25
0
0
25
50
75
100
125
150
175
200
225
Dose (μg/mL)

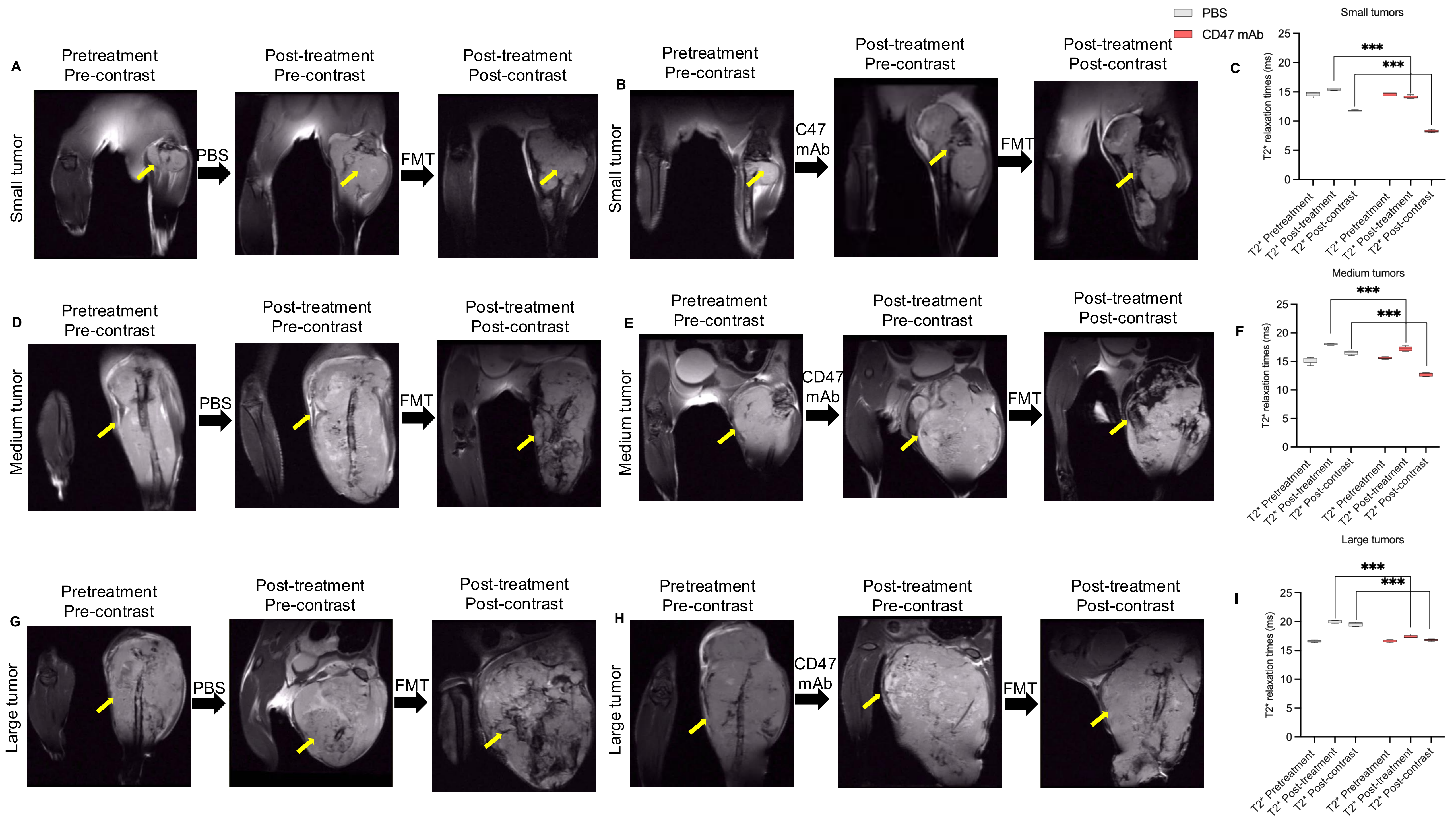
A
Small tumor
Pretreatment
Pre-contrast
PBS
Post-treatment
Pre-contrast
FMT
Post-treatment
Post-contrast
B
Small tumor
Pretreatment
Pre-contrast
C47
mAb
Post-treatment
Pre-contrast
FMT
Post-treatment
Post-contrast
C
PBS
CD47 mAb
Small tumors
T2* relaxation times (ms)
***
***
T2* Pretreatment
T2* Post-treatment
T2* Post-contrast
T2* Pretreatment
T2* Post-treatment
T2* Post-contrast
D
Medium tumor
Pretreatment
Pre-contrast
PBS
Post-treatment
Pre-contrast
FMT
Post-treatment
Post-contrast
E
Medium tumor
Pretreatment
Pre-contrast
CD47
mAb
Post-treatment
Pre-contrast
FMT
Post-treatment
Post-contrast
F
Medium tumors
T2* relaxation times (ms)
***
***
T2* Pretreatment
T2* Post-treatment
T2* Post-contrast
T2* Pretreatment
T2* Post-treatment
T2* Post-contrast
G
Large tumor
Pretreatment
Pre-contrast
PBS
Post-treatment
Pre-contrast
FMT
Post-treatment
Post-contrast
H
Large tumor
Pretreatment
Pre-contrast
CD47
mAb
Post-treatment
Pre-contrast
FMT
Post-treatment
Post-contrast
I
Large tumors
T2* relaxation times (ms)
***
***
T2* Pretreatment
T2* Post-treatment
T2* Post-contrast
T2* Pretreatment
T2* Post-treatment
T2* Post-contrast

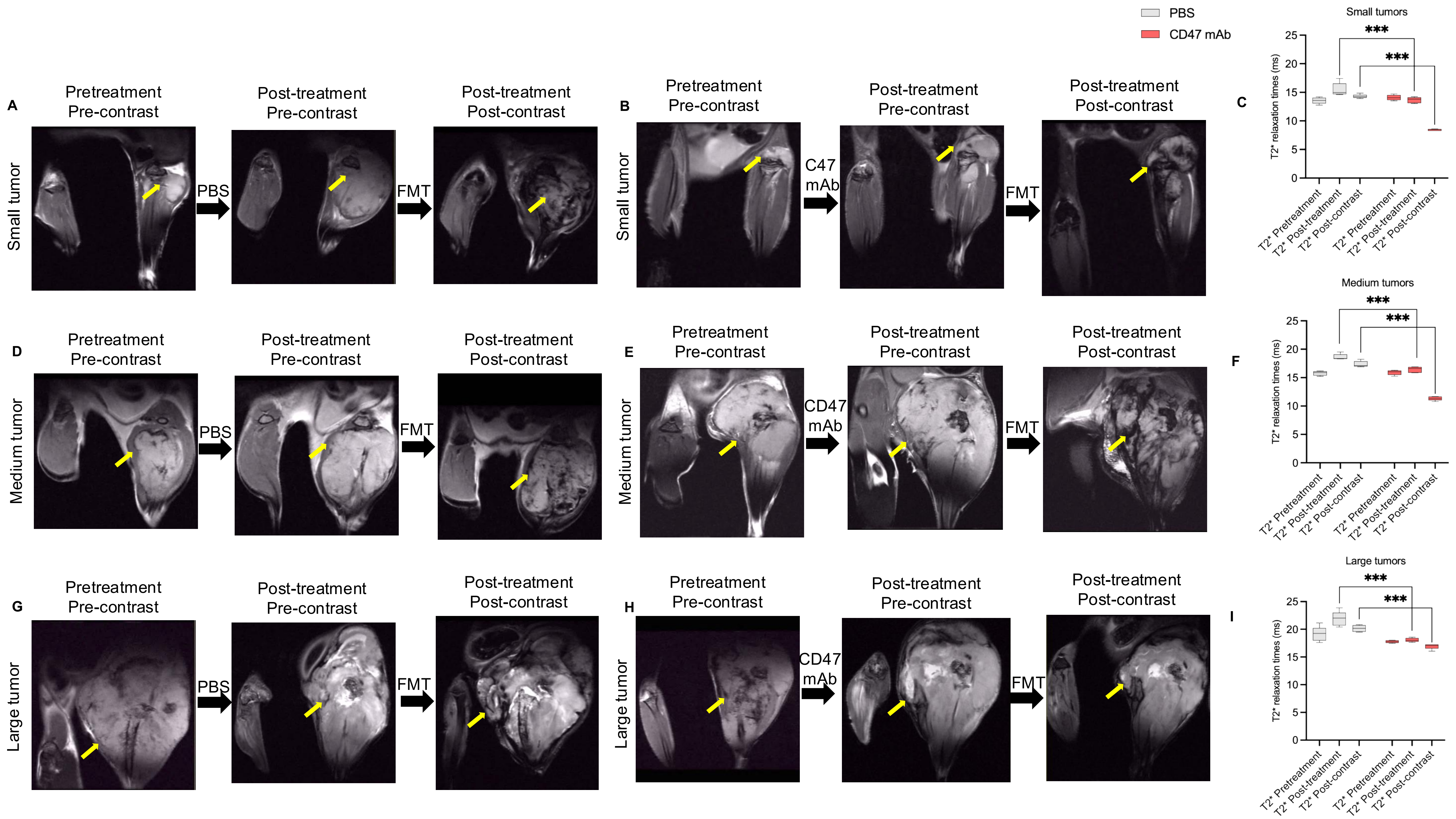

A
B
C
D
E
F
G
H
I
Pretreatment Pre-contrast
Post-treatment Pre-contrast
Post-treatment Post-contrast
Small tumor
Medium tumor
Large tumor
PBS
FMT
C47 mAb
CD47 mAb
PBS
CD47 mAb
Small tumors
Medium tumors
Large tumors
T2* relaxation times (ms)
T2* Pretreatment
T2* Post-treatment
T2* Post-contrast
***

A

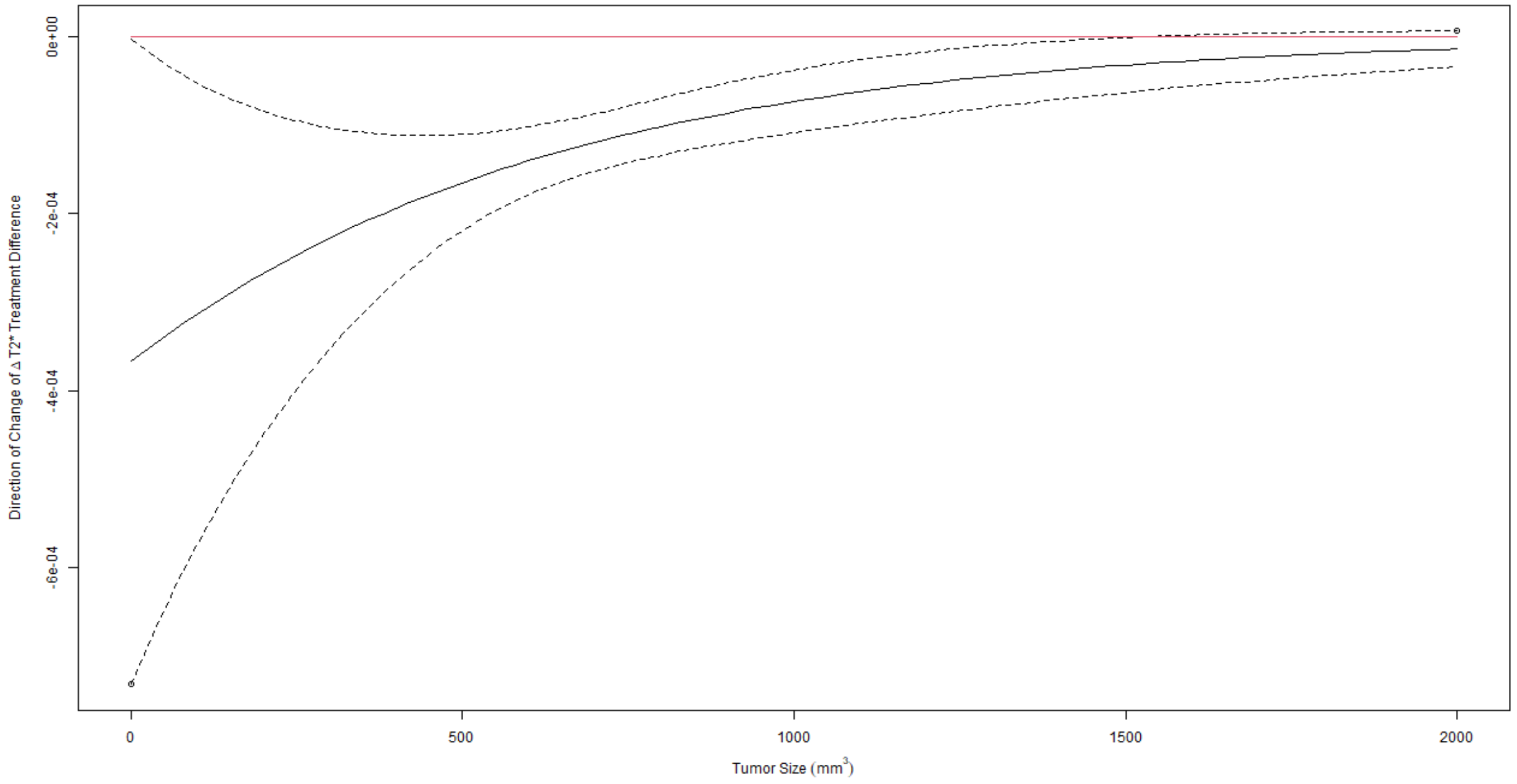
0e+00
-2e-04
-4e-04
-6e-04
Direction of Change of ∆ T2* Treatment Difference
0
500
1000
1500
2000
Tumor Size (mm3)

B

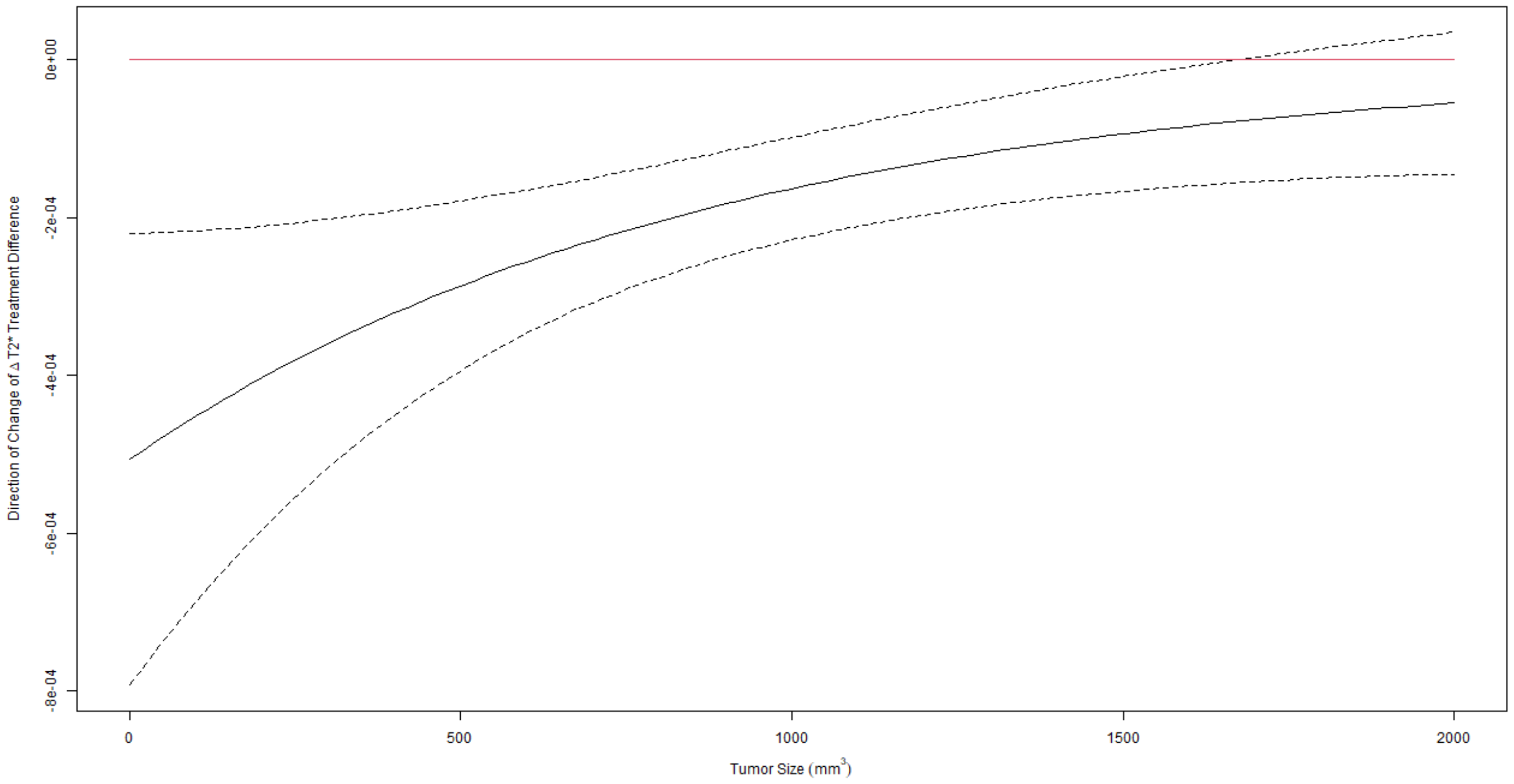
0e+00
-2e-04
-4e-04
-6e-04
-8e-04
Direction of Change of ∆ T2* Treatment Difference
0
500
1000
1500
2000
Tumor Size (mm3)

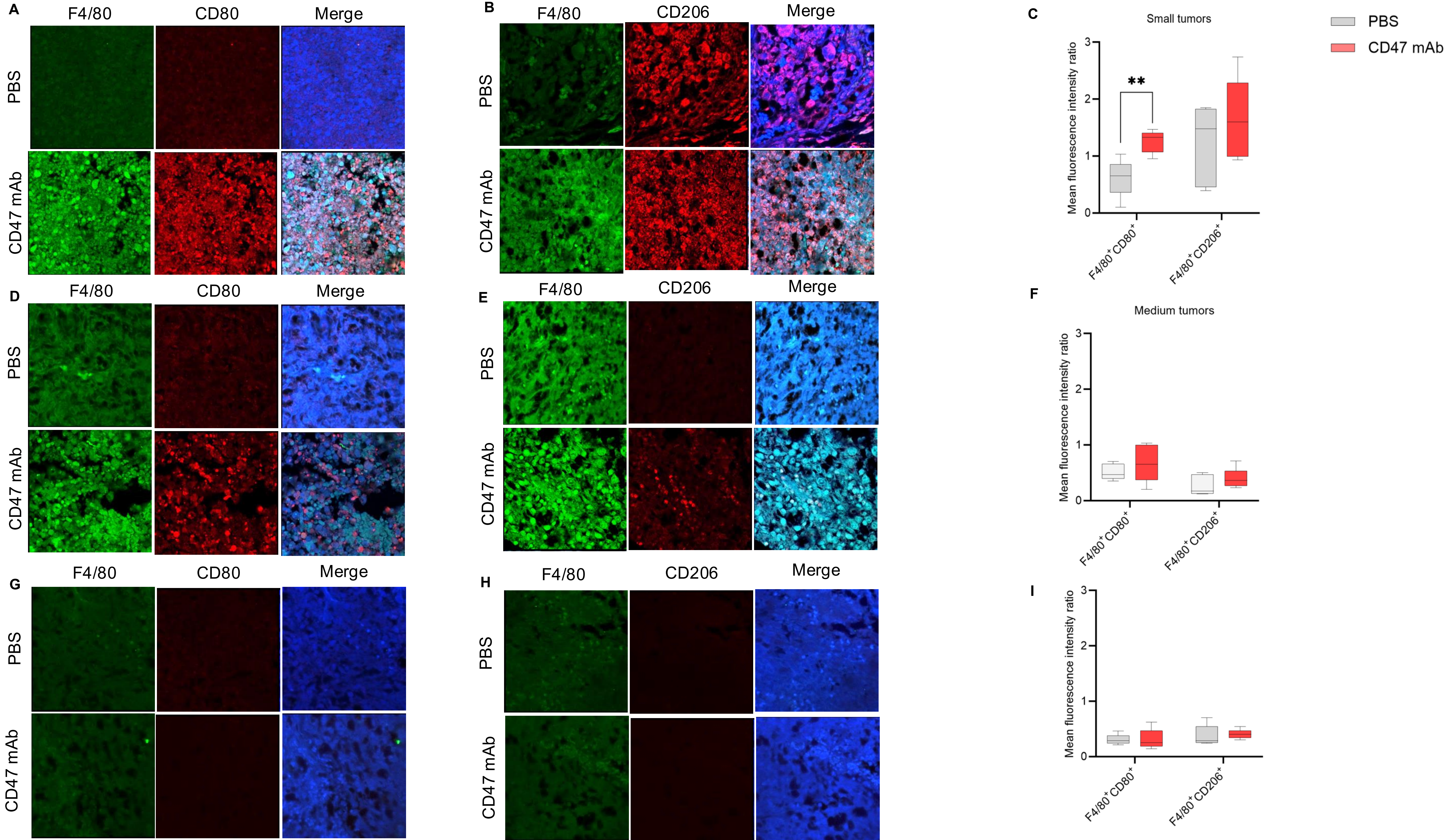
A
F4/80
CD80
Merge
PBS
CD47 mAb
B
F4/80
CD206
Merge
PBS
CD47 mAb
C
Small tumors
PBS
CD47 mAb
Mean fluorescence intensity ratio
**
F4/80+CD80+
F4/80+CD206+
D
F4/80
CD80
Merge
PBS
CD47 mAb
E
F4/80
CD206
Merge
PBS
CD47 mAb
F
Medium tumors
Mean fluorescence intensity ratio
F4/80+CD80+
F4/80+CD206+
G
F4/80
CD80
Merge
PBS
CD47 mAb
H
F4/80
CD206
Merge
PBS
CD47 mAb
I
Mean fluorescence intensity ratio
F4/80+CD80+
F4/80+CD206+

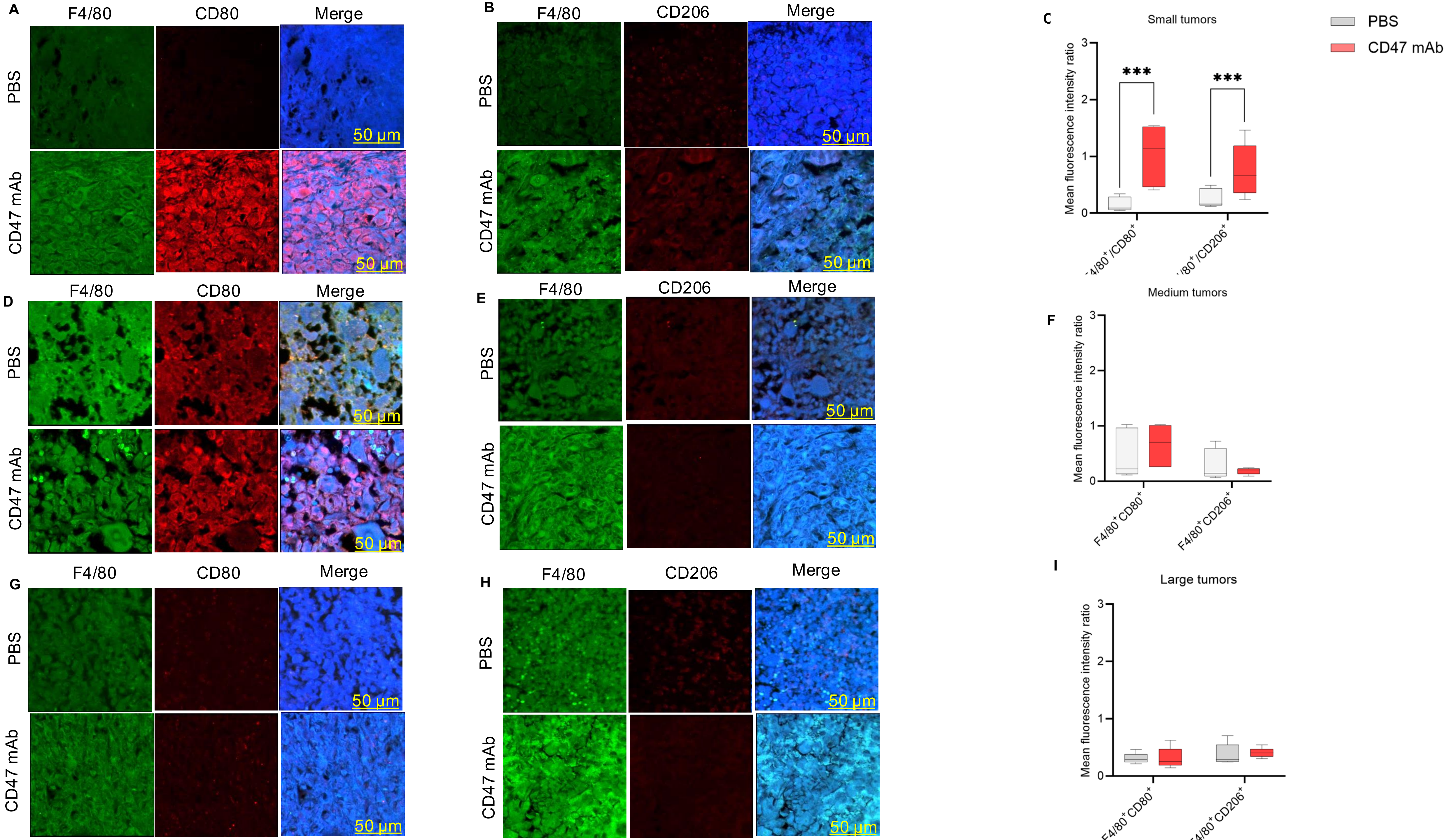

A
F4/80
CD80
Merge
PBS
CD47 mAb
50 µm
B
CD206
C
Small tumors
PBS
CD47 mAb
Mean fluorescence intensity ratio
***
D
E
F
Medium tumors
F4/80+CD80+
F4/80+CD206+
G
H
I
Large tumors

A

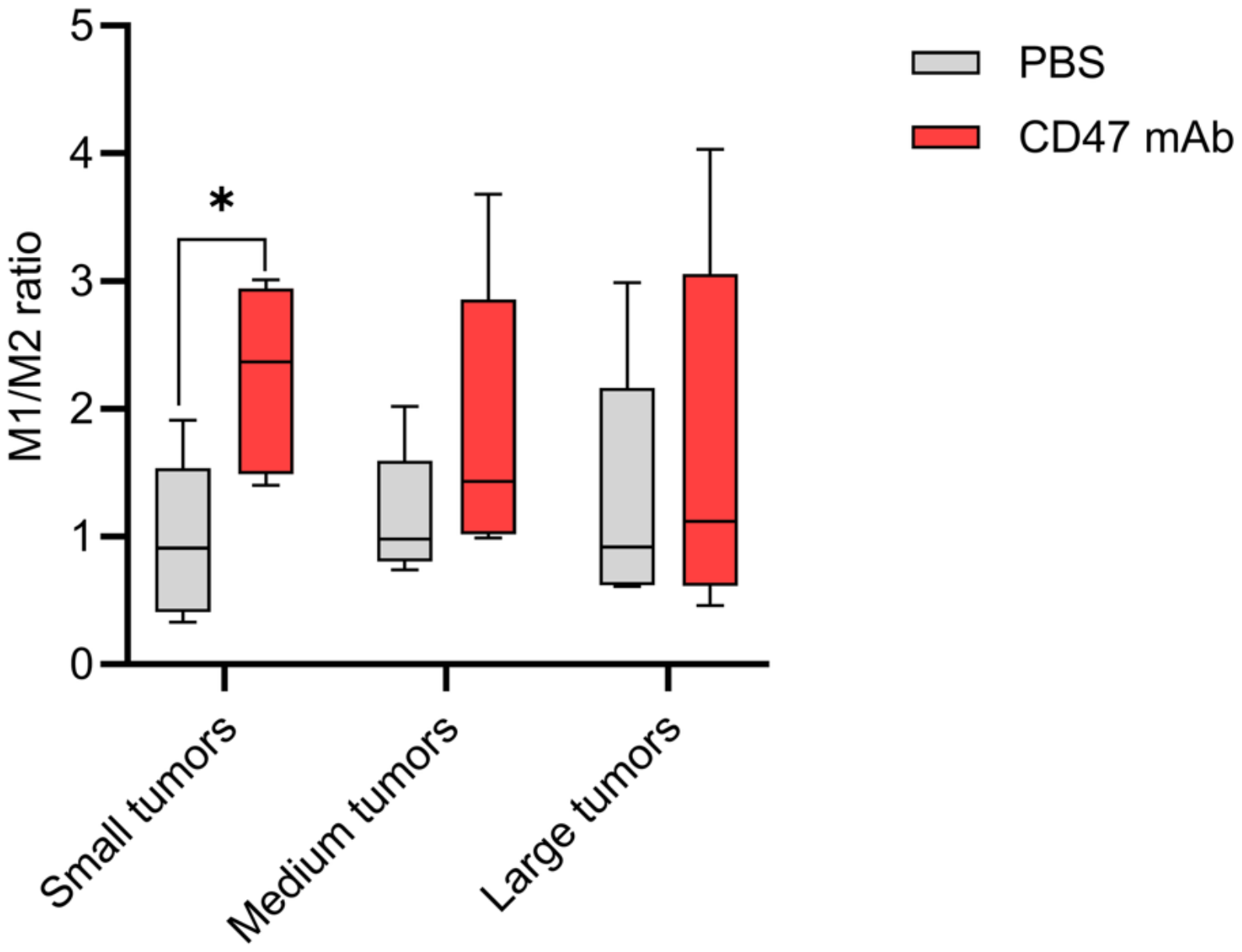

PBS
CD47 mAb
M1/M2 ratio
0
1
2
3
4
5
*
Small tumors
Medium tumors
Large tumors

B

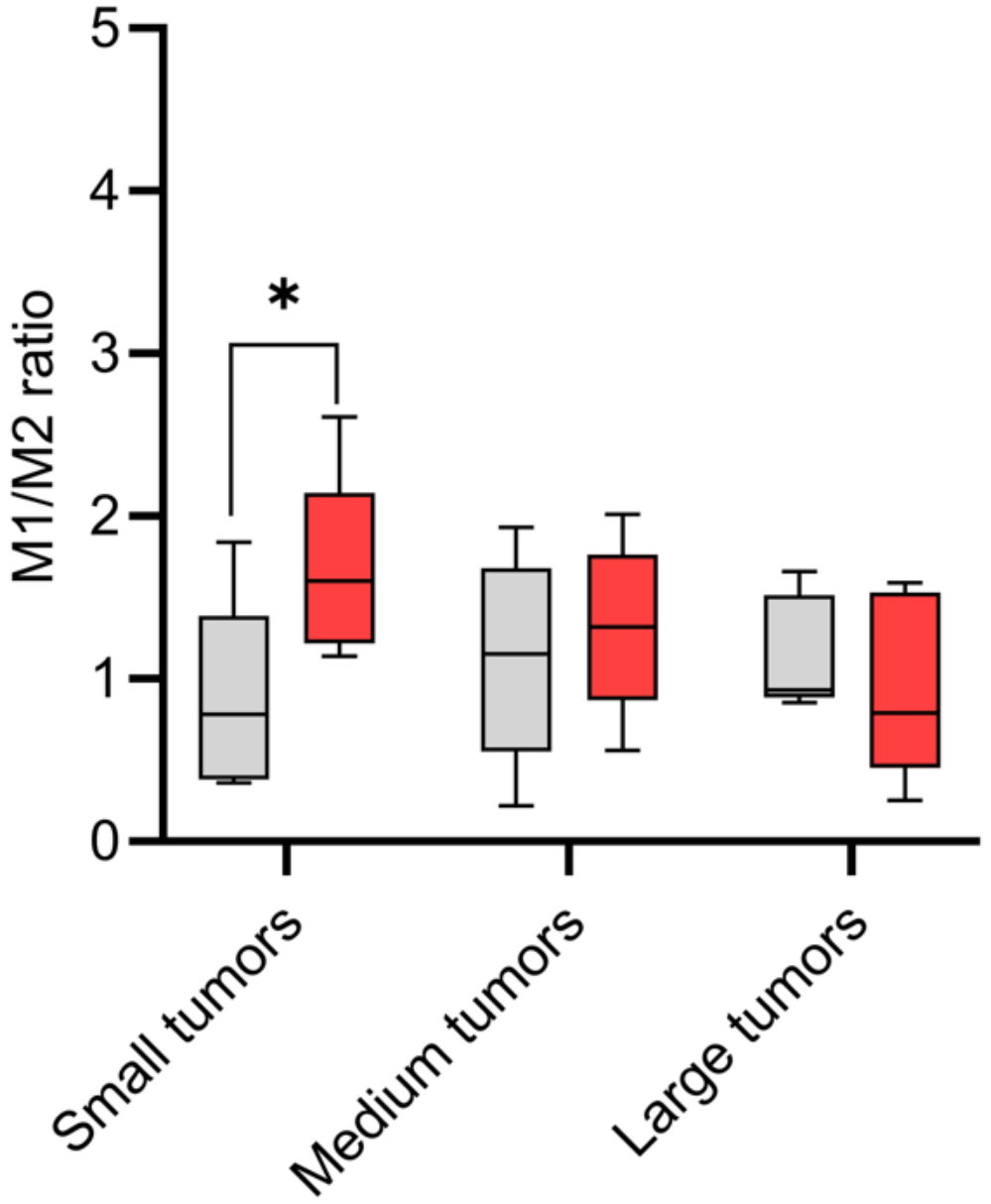

M1/M2 ratio
0
1
2
3
4
5
*
Small tumors
Medium tumors
Large tumors
