# Supplementary material for: Tumor size limits macrophage activation and imaging response to CD47 blockade in osteosarcoma
Source: Biomed Pharmacother. Author manuscript; Available in PMC 2026 Oct 28. (PMC13714959; doi:10.1016/j.biopha.2026.119846)
Supplement: Supplementary file 2 [file NIHMS2211656-supplement-1.docx]

**Supplementary File 1:**

**Comparison of T2* relaxation times between small and medium-sized human 143B osteosarcomas**

**Tumor T2* relaxation times in PBS-treated tumors**

At baseline (pretreatment, pre-contrast), tumor T2* relaxation times were not significantly different between small tumors (T2* = 14.61 ± 0.37 ms) and medium tumors (T2* = 15.24 ± 0.56 ms, p = 0.30), although medium tumors showed a trend toward slightly longer T2* values. After 7 days of PBS treatment (post-treatment, pre-contrast), T2* relaxation times were not significantly different between small tumors (T2* = 15.43 ± 0.22 ms) and medium tumors (T2* = 18.03 ± 0.16 ms, p=0.06). Within each size group, T2* values showed a trend toward increase after PBS treatment compared with baseline, consistent with tumor growth. After 7 days of PBS treatment and ferumoxytol injection (post-treatment, post-contrast), T2* values were not significantly different between small tumors (T2* = 11.76 ± 0.13 ms) and medium tumors (T2* = 16.49 ± 0.32 ms, p = 0.30).

**Tumor T2* relaxation times in CD47 mAb-treated tumors**

At baseline (pretreatment, pre-contrast), tumor T2* relaxation times were not significantly different between small tumors (T2* = 14.60 ± 0.31 ms) and medium tumors (T2* = 15.59 ± 0.18 ms, p = 0.30), with medium tumors showing a trend toward slightly longer T2* values. After 7 days of CD47 mAb treatment (post-treatment, pre-contrast), tumor T2* relaxation times remained similar to baseline values within each size group and were not significantly different between small tumors (T2* = 14.10 ± 0.26 ms) and medium tumors (T2* = 17.20 ± 0.42 ms, p = 0.06). After 7 days of CD47 mAb treatment and ferumoxytol injection (post-treatment, post-contrast), T2* values were not significantly different between small tumors (T2* = 8.27 ± 0.22 ms) and medium tumors (T2* = 12.75 ± 0.34 ms, p = 0.30).

**Comparison of T2* relaxation times between small and large murine K7M2 Osteosarcomas treated with CD47 mAb or PBS**

**Tumor T2* relaxation times in PBS-treated tumors**

At baseline (pretreatment, pre-contrast), tumor T2* relaxation times showed no significant differences between small tumors (T2* = 13.55 ± 0.56 ms) and large tumors (T2* = 19.13 ± 1.34 ms; p = 0.58). Following 7 days of PBS treatment (post-treatment, pre-contrast), T2* relaxation times were significantly different between small tumors (T2* = 15.47 ± 1.17 ms) and large tumors (T2* = 21.88 ± 1.32 ms; p = 0.01). Both groups showed a trend toward longer T2* values compared with baseline, indicative of tumor growth. After 7 days of PBS treatment and ferumoxytol injection (post-treatment, post-contrast), T2* values decreased in both small tumors (T2* = 14.29 ± 0.35 ms) and large tumors (T2* = 20.15 ± 0.60 ms). Notably, T2* relaxation times were significantly shorter in small tumors compared with large tumors (p < 0.001), suggesting a higher density of TAMs in small tumors.

**Tumor T2* relaxation times in CD47 mAb-treated tumors**

At baseline (pretreatment, pre-contrast), tumor T2* relaxation times did not differ significantly between small tumors (T2* =14.05 ± 0.48 ms) and large tumors (T2* = 17.78 ± 0.24 ms; p = 0.58), although large tumors showed a trend towards slightly longer T2* values. Following 7 days of CD47 mAb treatment (post-treatment, pre-contrast), T2* values remained similar to baseline within each size group but were significantly different between small tumors (T2* = 13.64 ± 0.52 ms) and large tumors (T2* =18.08 ± 0.37 ms; p = 0.01). After 7 days of CD47 mAb treatment and ferumoxytol injection (post-treatment, post-contrast), T2* values significantly decreased compared with pre-contrast in both small tumors (T2* = 8.41 ± 0.16 ms; p < 0.001) and large tumors (T2* = 16.93 ± 0.50 ms; p < 0.001). T2* relaxation times were significantly shorter in small tumors compared with large tumors (p < 0.001), indicating elevated phagocytic activity of TAMs in small tumors.

**Macrophage marker expression in murine osteosarcoma tumors**

Immunofluorescence analyses revealed increased staining for F4/80⁺, CD80⁺, and CD206⁺ TAMs in small tumors treated with CD47 mAb compared with PBS-treated controls (Figure S4). In contrast, TAM marker expression was lower in large tumors regardless of treatment. Quantitative analysis demonstrated a significantly higher number of F4/80⁺CD80⁺ cells in small tumors treated with CD47 mAb compared with PBS (p < 0.001), suggesting enhanced M1 macrophage activation. F4/80⁺CD206⁺ cells were also more abundant in small CD47 mAb-treated tumors compared with small PBS-treated tumors (p = 0.007). In contrast, large CD47 mAb-treated tumors and large PBS-treated tumors showed no significant differences in F4/80⁺CD80⁺ or F4/80⁺CD206⁺ cell counts, indicating limited TAM activation with high tumor burden (all p > 0.05).

Across tumor sizes, TAM marker expression was consistently higher in small tumors than in large tumors. Notably, F4/80⁺CD80⁺ staining was significantly higher in small tumors compared with large tumors following CD47 mAb treatment (p = 0.02).

**Supplementary figure legends**:

**Figure S1.** Effect of ferumoxytol on RAW 264.7 macrophage viability. RAW 264.7 macrophages were treated with increasing concentrations of ferumoxytol (25–200 µg/mL) for 24 h. Cell viability was measured as a percentage of untreated control (100% viability). Data represent mean ± SD (n = 3 independent experiments). No significant differences in cell viability were observed across the tested concentrations.

**Figure S2.** MRI of small, medium, and large 143B human osteosarcomas before and after treatment with PBS or CD47 mAb. (A, B) Representative coronal T2-weighted MRI of small 143B tumors treated with PBS (A) or CD47 mAb (B) at three time points: pretreatment (pre-contrast), post-treatment (pre-contrast), and post-treatment (post-contrast, 24 h after intravenous ferumoxytol injection). (C) T2* relaxation times (ms) of small tumors at three time points (D, E) Representative coronal T2-weighted MRI of medium 143B tumors treated with PBS (D) or CD47 mAb (E) at three time points. (F) T2* relaxation times (ms) of medium tumors at three time points. (G, H) Representative coronal T2-weighted MRI of large 143B tumors treated with PBS (G) or CD47 mAb (H) at three time points. (I) T2* relaxation times (ms) of large tumors at three time points. Data represent mean ± SD from five tumors per group. Only statistically significant comparisons are shown (*** p < 0.001).

**Figure S3.** MRI of small, medium, and large K7M2 murine osteosarcomas before and after treatment with PBS or CD47 mAb. (A, B) Representative coronal T2-weighted MRI of small K7M2 tumors treated with PBS (A) or CD47 mAb (B) at three time points: pretreatment (pre-contrast), post-treatment (pre-contrast), and post-treatment (post-contrast, 24 h after intravenous ferumoxytol injection). (C) T2* relaxation times (ms) of small tumors at three time points. (D, E) Representative coronal T2-weighted MRI of medium K7M2 tumors treated with PBS (D) or CD47 mAb (E) at three time points. (F) T2* relaxation times (ms) of medium tumors at three time points. (G, H) Representative coronal T2-weighted MRI of large K7M2 tumors treated with PBS (G) or CD47 mAb (H) at three time points. (I) T2* relaxation times (ms) of large tumors at three time points. Data represent mean ± SD from five tumors per group. Only statistically significant comparisons are shown (*** p < 0.001).

**Figure S4.** Size-dependent treatment effect of CD47 mAb versus PBS. Poisson regression models were fitted for CD47 mAb and PBS treatment groups. The derivative of the between-group difference in ΔT2* was estimated using resampling methods. A negative derivative indicates that the treatment effect decreases with increasing tumor size. For both 143B (A) and K7M2 (B) tumors, the 95% confidence interval of the derivative remained significantly negative (different from 0) up to tumor sizes of 1500 mm³, indicating that the treatment effect difference between CD47 mAb and PBS is detectable below this threshold.

**Figure S5:** Histopathological analysis of small, medium, and large human 143B osteosarcomas after PBS or CD47 mAb treatment. (A-C) Small tumors: Representative immunofluorescence confocal images (×20) of small tumors treated with PBS or CD47 mAb, stained for (A) F4/80⁺ and CD80⁺ (M1) or (B) F4/80⁺ and CD206⁺ (M2) TAM markers. (C) Mean fluorescence intensity of F4/80⁺CD80⁺ and F4/80⁺CD206⁺ TAMs. (D-F) Medium tumors: Representative immunofluorescence confocal images (×20) of medium tumors treated with PBS or CD47 mAb, stained for (D) F4/80⁺ and CD80⁺ (M1) or (E) F4/80⁺ and CD206⁺ (M2) TAM markers. (F) Mean fluorescence intensity of F4/80⁺CD80⁺ and F4/80⁺CD206⁺ TAMs. (G–I) Large tumors: Representative immunofluorescence confocal images (×20) of large tumors treated with PBS or CD47 mAb, stained for (G) F4/80⁺ and CD80⁺ (M1) or (H) F4/80⁺ and CD206⁺ (M2) TAM markers. (I) Mean fluorescence intensity of F4/80⁺CD80⁺ and F4/80⁺CD206⁺ TAMs. Data represent mean ± SD from five tumors per group. Only statistically significant comparisons are shown (** p < 0.01).

**Figure S6:** Histopathological analysis of small, medium and large murine K7M2 osteosarcomas after PBS or CD47 mAb treatment. (A–C) Small tumors: Representative immunofluorescence confocal images (×20) of small tumors treated with PBS or CD47 mAb, stained for (A) F4/80⁺ and CD80⁺ (M1) or (B) F4/80⁺ and CD206⁺ (M2) TAM markers. (C) Mean fluorescence intensity of F4/80⁺CD80⁺ and F4/80⁺CD206⁺ TAMs. (D–F) Medium tumors: Representative immunofluorescence confocal images (×20) of medium tumors treated with PBS or CD47 mAb, stained for (D) F4/80⁺ and CD80⁺ (M1) or (E) F4/80⁺ and CD206⁺ (M2) TAM markers. (F) Mean fluorescence intensity of F4/80⁺CD80⁺ and F4/80⁺CD206⁺ TAMs. (G–I) Large tumors: Representative immunofluorescence confocal images (×20) of large tumors treated with PBS or CD47 mAb, stained for (G) F4/80⁺ and CD80⁺ (M1) or (H) F4/80⁺ and CD206⁺ (M2) TAM markers. (I) Mean fluorescence intensity of F4/80⁺CD80⁺ and F4/80⁺CD206⁺ TAMs. Data represent mean ± SD from five tumors per group. Only statistically significant comparisons are shown (*** p < 0.001).

**Figure S7:** M1/M2 macrophage ratios in size-stratified osteosarcomas following CD47 blockade. M1/M2 macrophage ratios in (A) 143B and (B) K7M2 osteosarcomas. The intratumoral M1/M2 macrophage ratio was determined in small, medium, and large tumors after treatment with PBS or CD47 mAb. Data represent mean ± SD from five tumors per group. Only statistically significant comparisons are shown (* p < 0.05).
